# Altered neural odometry in the vertical dimension

**DOI:** 10.1073/pnas.1811867116

**Published:** 2019-02-15

**Authors:** Giulio Casali, Daniel Bush, Kate Jeffery

**Affiliations:** ^a^Department of Cell and Developmental Biology, University College London, London WC1E 6BT, United Kingdom;; ^b^Institute of Cognitive Neuroscience, University College London, London WC1N 3AZ, United Kingdom;; ^c^Institute of Neurology, University College London, London WC1N 3BG, United Kingdom;; ^d^Department of Experimental Psychology, University College London, London WC1H 0AP, United Kingdom

**Keywords:** hippocampus, entorhinal cortex, grid cells, place cells, three-dimensional space

## Abstract

Entorhinal grid cells integrate sensory and self-motion inputs to provide a spatial metric of a characteristic scale. One function of this metric may be to help localize the firing fields of hippocampal place cells during formation and use of the hippocampal spatial representation (“cognitive map”). Of theoretical importance is the question of how this metric, and the resulting map, is configured in 3D space. We find here that when the body plane is vertical as rats climb a wall, grid cells produce stable, almost-circular grid-cell firing fields. This contrasts with previous findings when the body was aligned horizontally during vertical exploration, suggesting a role for the body plane in orienting the plane of the grid cell map. However, in the present experiment, the fields on the wall were fewer and larger, suggesting an altered or absent odometric (distance-measuring) process. Several physiological indices of running speed in the entorhinal cortex showed reduced gain, which may explain the enlarged grid pattern. Hippocampal place fields were found to be sparser but unchanged in size/shape. Together, these observations suggest that the orientation and scale of the grid cell map, at least on a surface, are determined by an interaction between egocentric information (the body plane) and allocentric information (the gravity axis). This may be mediated by the different sensory or locomotor information available on a vertical surface and means that the resulting map has different properties on a vertical plane than a horizontal plane (i.e., is anisotropic).

## Significance

The brain’s spatial map is supported by place cells, encoding current location, and grid cells, which report horizontal distance traveled by producing evenly sized and spaced foci of activity (firing fields) that tile the environment surface. We investigated whether the metric properties of the cells’ activity are the same in vertical space as in horizontal. On a vertical wall, grid-cell firing fields were enlarged and more widely spaced, while place-cell firing fields were unchanged in size/shape but less prevalent. Sensitivity of single-cell and population field potential activity to running speed was reduced. Together, these results suggest that spatial encoding properties are determined by an interaction between the body-plane alignment and the gravity axis.

Self-localization, a fundamental cognitive skill, relies on the integration of self-motion cues (path integration) with external information processed by the sensory systems (1). Accumulating evidence suggests that at least part of this process takes place in the entorhinal grid cell system, which interacts with place cells in the hippocampus (HPC) to maintain a representation of both current location and distance traveled. These parameters are reflected in the activity of place cells, which, in small spaces, produce mostly single foci of firing (2) called “firing fields”, and grid cells, which produce a grid-like array of firing fields spread across the environment (3–5). When animals locomote on a horizontal surface, both place cells and grid cells use environmental landmarks to establish or correct location estimates, together with self-motion cues, such as running speed and angular velocity, to update those estimates (6).

Real-world environments tend to be nonhorizontal much of the time, and, in such environments, the animal’s head is itself not always horizontal either, which alters the sensory processing of self-motion. An important question is thus whether sensory integration by place and grid cells works in the same way in three dimensions as it does in two. Studies of flying bats have suggested that place and head direction cells have similar properties in volumetric space (7, 8), but a study in rats suggested that grid cells might not compute distance traveled in the vertical dimension (9). In that study, rats explored a vertical peg-studded wall (the “pegboard”) or climbed a helical staircase: grid cells were found to show periodicity in the horizontal component of their movement but not the vertical, and place cells produced firing fields that were elongated in the vertical dimension; both observations suggesting reduced/absent odometry in the vertical dimension. However, while climbing these structures the animals maintained a horizontally aligned body plane, and so the grid plane might have been specified either by gravity or by the body plane of the animal, or both. Subsequently, on a steep (40 deg.) slope, grid cells were shown to exhibit similar firing patterns to those observed on a horizontal surface (10). Together, these studies suggest that perhaps grid cells perform odometry in the plane of locomotion (the surface the feet are on) regardless of its orientation in 3D space, and not in the direction orthogonal to that plane.

To address this question, we recorded place and grid cells as rats with extensive climbing experience foraged freely over horizontal and vertical surfaces with their body axis always oriented parallel to the foraging surface. If the grid plane is defined by gravity then grid cell encoding on the vertical wall should produce “stripes” (i.e., be anisotropic), as previously observed (9). Conversely, if the grid plane is defined by the animal’s body plane then firing fields should also be grid-like (circular, and evenly spaced) on the wall. Because of the importance of self-motion signals to grid cell odometry, we also recorded “speed cells” (11) and local field potentials (LFPs), both of which have been implicated in speed encoding (12) and grid cell odometry (13, 14).

## Results

**Fig. 1.**
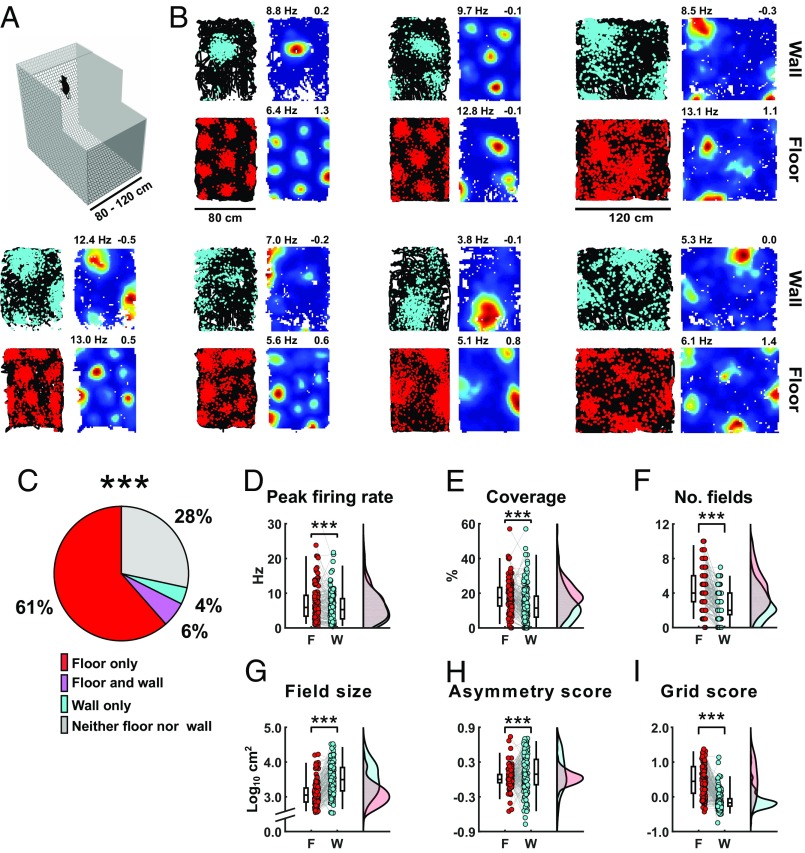
Spatial firing patterns of grid cells on the floor vs. the wall. (*A*) Schematic representation of the floor-wall apparatus, with the side walls shown as transparent for clarity. (*B*) Examples of the firing patterns of seven grid cells from six rats on floor (*Bottom Left*, red) and wall (*Top Left*, turquoise). For each cell, the *Left* shows the animal’s path (black lines) with spikes (colored dots) superimposed, and the *Right* shows firing-rate heat maps from red (maximum) to blue (zero). Values above the heat maps show the peak firing rate (at left) and grid score (at right). (*C*) There was a significant drop in the number of grid cells on the wall compared with the floor, as shown in the pie chart representing percentages of grid cells (*n* = 148) that reached classification criteria on each of the two surfaces. (For the full classification, including the open field, see *SI Appendix*, Fig. S4*O*.) (*D*–*I*) Firing parameters between surfaces: box plots (outside) and colored points (inside) connected by gray lines representing the same cells on floor (red) and wall (turquoise) (*Left*) and rain plots showing kernel density estimate of firing parameters across surfaces (*Right*). The code for raincloud plot visualization has been adapted from Allen et al. (23).

We recorded 148 unique grid cells from the medial entorhinal cortex (mEC) of 11 rats and 72 place cells from the HPC of three rats (*SI Appendix*, Fig. S1), all familiar with 3D environments (see *Methods*), as they foraged in the open field, or over a floor and adjoining wall (Fig. 1*A*). Of the grid cells, 72 cells met the grid score criterion on only one of the two horizontal surfaces. In the majority of cases, the discrepancy occurred because the cell had a slightly less regular grid (*n* = 52) or absent grid (*n* = 6) on one of the surfaces. In a few cases (*n* = 14), the grid score was low on both surfaces and may have passed threshold by chance. We retained these cells so as not to artificially inflate the horizontal-surface grid scores. In addition to the grid cells, we recorded 1,497 nonspatial mEC neurons and LFPs from 48 sessions. Rats moved freely over the wall in all directions (*SI Appendix*, Fig. S2*A*), although running speed was lower on the wall, except when the rat was climbing upward (*SI Appendix*, Fig. S2*B*), which was the easiest direction for them to manage.

### Grid-Cell Firing Patterns.

Entorhinal-cell firing properties were altered on the wall (Fig. 1*B* and *SI Appendix*, Fig. S3): fewer met the grid cell criteria (Fig. 1*C* and *SI Appendix*, Fig. S4*O*; floor = 100/148; wall = 15/148, McNemar’s test, χ^2^ = 72.7, *P* < 0.00001), and there was a reduction in both the mean firing rate (*SI Appendix*, Fig. S4*A*; floor = 1.4 ± 0.1 Hz, wall = 1.1 ± 0.1 Hz, *t*_147_ = 6.89, *P* = 1.48 × 10^−10^) and peak firing rate (Fig. 1*D*; floor = 7.1 ± 0.4 Hz, wall = 5.9 ± 0.3 Hz, *t*_147_ = 3.98, *P* = 0.0001). The most striking observation was that on the wall, unlike on the pegboard (9), grid cells produced discrete firing fields rather than stripes (Fig. 1*B* and *SI Appendix*, Fig. S3). Nonetheless, grid-cell firing patterns differed significantly between surfaces. While grid cells showed no differences in spatial coherence (*SI Appendix*, Fig. S4*D*) and reduced stability (*SI Appendix*, Fig. S4*D*), firing fields on the wall covered less of the surface (Fig. 1*E* and *SI Appendix*, Fig. S4*F*; coverage: floor = 18.1 ± 0.7%, wall = 13.0 ± 0.8%, *t*_147_ = 5.62, *P* = 8.87 × 10^−8^), were fewer in number (Fig. 1*F* and *SI Appendix*, Fig. S4*F*; floor = 4.3 ± 0.2, wall = 2.6 ± 0.1, *t*_147_ = 8.89, *P* = 2.09 × 10^−15^), enlarged (Fig. 1*G* and *SI Appendix*, Fig. S4 *G* and *J*; field size; floor = 1840 ± 200 cm^2^, wall = 5348 ± 487 cm^2^, *t*_108_ = −7.29, *P* = 1.72 × 10^−11^), less symmetric [more elliptic (Fig. 1*H* and *SI Appendix*, Fig. S4 *H* and *K*); asymmetry score: floor = 0.000 ± 0.016, wall = 0.113 ± 0.027, *t*_147_ = −3.88, *P* = 0.0002], and showed no evidence of sixfold symmetry on the wall (Fig. 1*I* and *SI Appendix*, Fig. S4 *L*; grid score: floor = 0.48 ± 0.03, wall = −0.11 ± 0.02, *t*_147_ = 13.3, *P* = 6.19 × 10^−27^).

In addition, we explored whether the decline in the overall grid score on the wall could be an artifact of the concomitant reduction in the number of fields. Unpaired comparisons between grid scores of cells equated for the number of fields (one to seven fields) on both surfaces confirmed the reduced grid score on the wall for matched cells having one to four and six fields (*SI Appendix*, Fig. S4*M*). Similarly, we also found a reduced proportion of grid cells with a significant grid score on the wall when comparing between cells with equal number of fields (*SI Appendix*, Fig. S4*N*), using both a temporal and a spatial shuffling approach to obtain a null distribution.

Together, the findings reveal two key properties of the grid-cell firing pattern. First, the presence of discrete firing fields instead of vertical stripes (9) shows that grid cell firing is modulated by the orientation of the animal’s locomotion plane; second, differences in grid cell properties between the floor and wall show that grid-cell firing patterns differ across dimensions (i.e., are anisotropic).

### Place-Cell Firing Patterns.

**Fig. 2.**
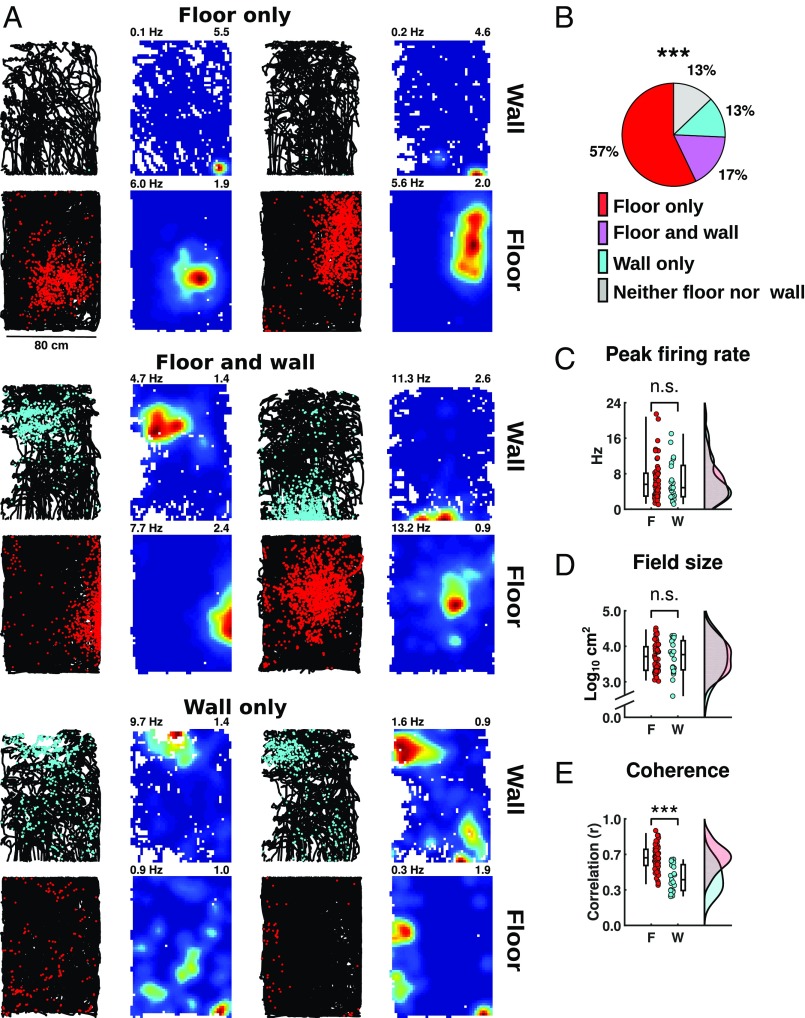
Preserved spatial metrics of place cells on the wall. (*A*) Examples of the firing patterns of six place cells, as shown in Fig. 1, classified as active: on the floor only (*Top* row), on floor and wall (*Middle* row), and on the wall only (*Bottom* row). (*B*) Pie chart showing the percentage of (*n* = 72) place cells active on each surface (color code as in Fig. 1*C*), illustrating a significant drop in the number of active cells on the wall compared with the floor. (*C*–*E*) Comparison of firing parameters between surfaces, as shown in Fig. 1.

The enlarged spatial maps exhibited by grid cells on the wall raise the question of whether place cell activity and/or place field metrics would also be affected. In contrast to grid cells, which fired on every surface, we found that fewer hippocampal place cells (*n* = 72; Fig. 2*A* and *SI Appendix*, Fig. S5) were active on the wall (Fig. 2*B* and *SI Appendix*, Fig. S6*I*; floor = 52/70, wall = 21/70; McNemar’s test, χ^2^= 18.4, *P* = 1.82 × 10^−5^). However, metric analysis of the place fields revealed few differences between floor and wall: unpaired comparisons between cells active on either surface found no difference in mean rates (*SI Appendix*, Fig. S6*A*) or peak firing rates (Fig. 2*C* and *SI Appendix*, Fig. S6*B*; floor = 6.4 ± 0.6 Hz, wall = 6.3 ± 1.0 Hz, *t*_71_ = 0.15, *P* = 0.88), place field size (Fig. 2*D* and *SI Appendix*, Fig. S6*C*; floor = 7626 ± 1023 cm^2^, wall = 7802 ± 1504 cm^2^, *t*_71_ = −0.09, *P* = 0.92), spatial information (*SI Appendix*, Fig. S6*D*) asymmetry score (*SI Appendix*, Fig. S6*E*), or stability (*SI Appendix*, Fig. S6*F*). However, on the wall, there was a decrease in the coverage (*SI Appendix*, Fig. S6*G*) and coherence (Fig. 2*E*; floor = 0.63 ± 0.02, wall = 0.44 ± 0.03, *t*_71_ = 6.12, *P* = 4.68 × 10^−8^) of place-cell firing fields.

Together, these findings suggest that place cells differ from grid cells in that the effect of locomoting on the wall is to reduce the probability but not the metric properties of their firing.

### Speed-Coding Analyses.

We next sought to explain why grid-cell firing fields might be expanded on the wall. Two major classes of models have been proposed to explain grid-cell firing patterns: (*i*) continuous attractor network (CAN) models, which posit that grid firing patterns are generated by synaptic interactions between cells, and (*ii*) oscillatory interference (OI) models, which propose that grid firing patterns are generated by the summation of multiple velocity-controlled oscillator inputs (15, 16). Both classes of models require a running-speed signal to maintain spatially stable grid firing patterns, and so we hypothesized that the expanded grid-cell firing patterns on the wall could result from underestimation of movement speed. Hence, we predicted that the movement speed input to grid cells (11) would show reduced gain on the wall.

**Fig. 3.**
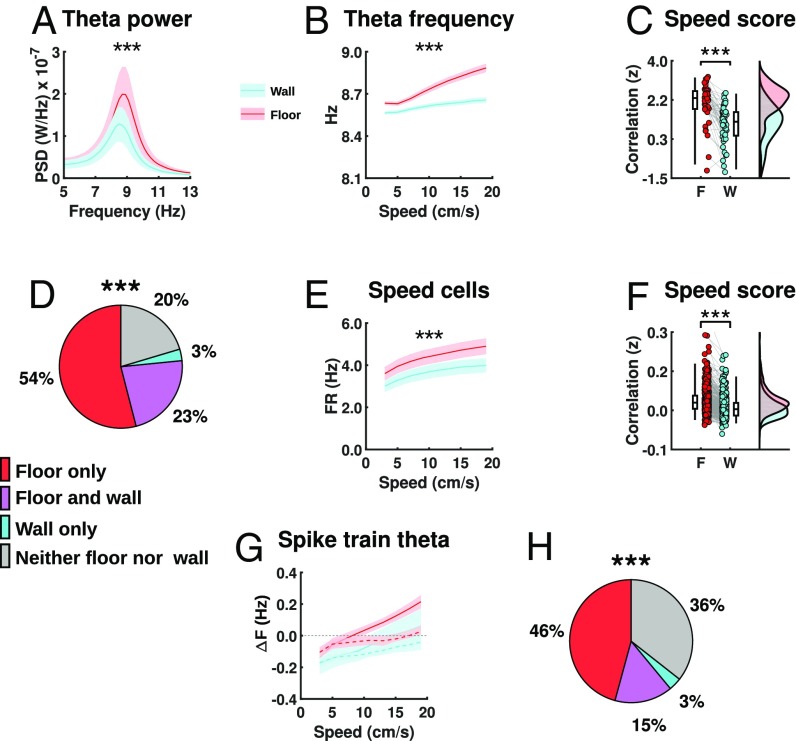
Altered speed coding on the wall. (*A*–*C*) Effects of vertical locomotion on LFP theta (*n* = 48). (*A* and *B*) Means (lines) ± SEM (shaded areas) between surfaces (floor, red; wall, turquoise). (*A*) Power spectrum density (PSD) showing clear peaks in the theta band (7–11 Hz) of both surfaces but reduced power and frequency on the wall compared with the floor. (*B*) Theta frequency as a function of running speed showing clear reduction on the wall compared with the floor. (*C*) Fisher’s Z transformation of the speed/theta frequency relationship between surfaces (shown as in Fig. 1). (*D*–*F*) Effects of vertical locomotion on speed cell firing. (*D*) There was a significant drop in the number of significant speed cells on the wall compared with the floor, as shown in the pie chart representing percentages of speed cells (*n* = 461) that reached classification criteria on each surface (color code as in Fig. 1*C*). Speed cells showed overall reduced firing rate (FR) across speed (*E*) and reduced speed score on the wall compared with the floor (Fisher’s Z transformation of the speed/firing rate relationship between surfaces) (*F*). (*G* and *H*) Effects of vertical locomotion on spiking rhythmicity of grid and speed cells. (*G*) Difference between mean spike train theta frequency and ΔF of grid cells (solid line) vs. speed cells (dashed line) between surfaces (floor, red; wall, turquoise) across speed. (*H*) There was a significant drop in the number of PPP grid cells on the wall compared with the floor, as shown in the chart representing percentages of rhythmic grid cells (*n* = 59) that reached PPP classification criteria on each surface (color code as in Fig. 1*C*).

In agreement with this prediction, both measured neural correlates of running speed—that is, LFP theta oscillations (12, 15) and the firing rate of speed cells (11)—exhibited altered relations with running speed on the wall. Spectral analysis of LFP theta showed reduction in both mean power and frequency (Fig. 3 *A* and *B* and *SI Appendix*, Fig. S7 *A* and *B*), due to a significant reduction in the slope (*SI Appendix*, Fig. S7*C*) but not the intercept (*SI Appendix*, Fig. S7*D*) of the running speed–theta frequency relationship (Fig. 3*B*), as well as a reduced correlation between the two (Fig. 3*C*; mean Pearson’s *r*: floor = 0.87 ± 0.05, wall = 0.60 ± 0.07; Fisher’s Z transformation, *t*_47_ = 8.92, *P* = 1.11 × 10^−11^).

In addition, fewer units met the criteria to be classified as speed cells on the wall (Fig. 3*D* and *SI Appendix*, Fig. S8*I*; floor = 353/461, wall = 118/461, McNemar’s test, χ^2^ = 208.8, *P* = 6.99 × 10^−19^), and those cells had reduced firing rates across all running speeds (Fig. 3*E* and *SI Appendix*, Fig. S8 *A*–*C*) alongside reduced speed scores (Fig. 3*F* and *SI Appendix*, Fig. S8 *D* and *E*; floor = 0.11 ± 0.003, wall = 0.067 ± 0.003, *t*_460_ = 19.8, *P* = 1.69 × 10^−63^).

Finally, because the frequency relationships of neuronal oscillations are important in the OI model, we examined spiking rhythmicity of grid and speed cells (17). We found fewer rhythmic cells on the wall (*SI Appendix*, Fig. S9 *A* and *B*). For cells that were rhythmic on both surfaces (grid = 95/148, speed = 277/459), we investigated whether phase precession (18)—thought to provide a temporal code for both location (19) and movement direction (20, 21)—persisted during climbing on the wall. To do so we took the difference between spike train and LFP theta frequency (ΔF) across a matched range of running speeds (2–20 cm/s) as a signature of theta phase precession (18, 19, 22–25). We found that grid cells, but not speed cells, showed positive ΔF (Fig. 3*G*) on the floor, as well as reduced theta phase-locking compared with speed cells (*SI Appendix*, Fig. S9*C*). Conversely, both grid and speed cells tended to have negative ΔF on the wall (Fig. 3*G* and *SI Appendix*, Fig. S9 *D* and *E*), consistent with a significant decrease in the number of putative phase-precessing (PPP) grid cells on the wall compared with the floor (Fig. 3*H* and *SI Appendix*, Fig. S9*F*; floor = 36/59, wall = 11/59; McNemar’s test, χ^2^ = 19.9, *P* = 8.32 × 10^−6^). Thus, it seems that the encoding of speed during climbing by both speed-cell firing rate and LFP theta frequency was underestimated, and the relationship between theta and spiking was altered.

## Discussion

The core question that motivated this study was whether the reference plane for the grid cell spatial metric is the horizontal plane (i.e., the Earth’s surface, perpendicular to gravity), the locomotor plane (i.e., the current walking surface, which may not be horizontal), or both. We found that although grid cells formed relatively circular firing fields on the wall, these were larger, slightly vertically elongated and may have been irregularly arranged (although the latter was difficult to confirm). In addition, we found that two principal electrophysiological signatures of running speed showed reduced gain during movement on the wall. Collectively, our findings suggest that grid cell odometry is weakly present during locomotion in the vertical plane but altered in scale, and also the observed increase in scale may be due to a reduction in the gain of speed signals in the mEC. Overall, we can conclude that although grid cell odometry is referenced to the locomotor surface, this is modulated by the orientation of that surface relative to gravity, revealing an interaction between egocentric and allocentric reference frames in shaping the spatial map. Below, we examine these observations and their implications for how 3D space is encoded in the brain.

### Grid Cell Odometry Was Present but Altered on the Wall.

The evidence that grid cells perform odometry at all on the wall is that they produced approximately circular and spatially separated firing fields, indicating at least some ability for the cells to process distance traveled and thus avoid producing contiguous or irregularly shaped fields. Because the number of firing fields on the wall generally fell below three, it was difficult to tell whether the grid pattern had simply expanded or whether the regular arrangement of firing fields had been lost altogether—we think it was at least sometimes the latter, due to the fact that sometimes the area of nonfiring seemed to exceed what would be expected based on field size (see examples in *SI Appendix*, Fig. S3), but the expansion and consequent reduction in field number and grid score make it hard to confirm this quantitatively.

The observation of odometry is significant because previous research found that grid cell odometry appeared to be entirely absent in the vertical dimension when the animals roamed over a pegboard climbing wall (9). One notable difference between these experiments is that on the pegboard, the animal’s body axis remained mostly horizontally aligned as it moved between the pegs, while in the present experiment, it remained aligned vertically, suggesting that the body plane might be the reference plane for movement tracking. Thus, body alignment may determine the reference plane, with the stripes appearing on the pegboard aligned in the direction orthogonal to the animal’s locomotor plane. However, as discussed below, there are other possibilities related to the different locomotor patterns on the various surfaces.

### Possible Causes of the Altered Grid Pattern.

The location of grid-cell firing fields is thought to depend on a combination of sensory anchoring to locations in the environment, together with self-motion information that shapes and spaces the fields appropriately. One possible source of sensory anchoring is the place cells, which project to grid cells via feedback to entorhinal layer V and might have a role in grid determination (15), which could explain how grids become anchored to familiar environmental cues (26). The idea that place cells provide input to grid cells is supported by experimental findings that place cell inactivation abolishes grids (27), while grid decoherence only affects place fields far from boundaries (14, 28–30). In this light, it is interesting that in the present experiment, there was a significant drop in the number of active place cells on the wall, which may partly underlie the reduced number of grid fields.

Self-motion inputs were also likely disrupted on the wall. Self-motion computation relies on a mix of locomotor (efference copy) cues and feedback from a combination of optic (or other sensory) flow, proprioceptive signals, and vestibular signals. Active locomotion (generated by motor commands) may be important for grid generation, since grids were found to be absent in passively transported rats despite the presence of all other cues to movement except proprioception (31). One possible reason for the vertical stripes on the pegboard in the experiment by Hayman et al. (9) is absence of active, or at least sustained, locomotion in the vertical dimension relative to the horizontal. In the present experiment in which the rats climbed the chicken wire surface, rats found it easier to run upward on the wall than either sideways or downward, as was evident both by eye and also in the speed/direction distribution analyses (*SI Appendix*, Fig. S2), so altered locomotor inputs could in principle be a factor in the grid disruption. Supporting this notion, we also found slight alterations in grid parameters such as field density between the two horizontal surfaces (*SI Appendix*, Fig. S4), one of which was covered in chicken wire, which may have affected locomotor parameters such as footfall-planning or attention. Furthermore, on the wall, the animals often crabbed sideways, producing a dissociation between head direction and travel direction, which might in principle affect grid coding (32). However, arguing against this explanation, grid field size across different heading directions did not differ from the upward direction, in which the locomotion pattern most resembled that on the floor.

An alternative explanation for the altered grid pattern is altered sensory (as opposed to motor) feedback about self-motion. Since previous experiments have found relatively normal head direction tuning on walls (33, 34), we focused on the linear component of the self-motion signal, which is assumed to be determined by a speed signal. We investigated speed signaling by recording LFPs and speed cell activity as a function of running speed and found that three speed-correlated electrophysiological parameters were altered on the wall. The observation that general grid field expansion occurred concomitantly with these blunted speed signals on the wall is consistent with both CAN and OI classes of models, which both predict expansion if the speed/theta relationship is flattened. While both classes of models account for the metric periodic firing of grid cells, they both need sensory input to reset and stabilize the grid from drifts due to accumulating path integration error. So, one possibility is that while the sensory input—perhaps via place cell feedback—enables spatial firing of grid cells on the wall (i.e., enlarged firing fields), the metric properties of grid cells are altered due to disrupted path integration mechanisms.

### Possible Causes of an Altered Speed Signal.

If a disrupted speed signal underlies the grid alteration, what causes this disruption? One possibility is that the altered locomotion pattern results in reduced motor efference signals reaching the system, lowering external drive to the grid cells. A second possibility is related to the sensory detection of speed of movement, one source of which is the vestibular system. Particularly important for linear speed detection are the otolith organs of the inner ear, which process both movement-related linear acceleration and gravity. Normally, when the head is in its canonical horizontal posture, the brain can separate the component of acceleration due to movement from that due to gravity (35). It may be that when the head is aligned vertically, this process no longer works well, or at least not in the same way, leading to an altered or perhaps even absent acceleration signal. Consistent with this hypothesis, recordings in virtual reality, when vestibular signals are also absent, produce expanded grid fields (36). Supporting the vestibular hypothesis more generally, pharmacological inactivation of the vestibular system has been found to affect the power and speed modulation of the LFP theta in the mEC (37), as well as place cell-encoding specificity (38). There may, however, be other sources of the disruption—perhaps animals were less attentive to optic flow and other cues due to the cognitive demands of locomoting on the wall, perhaps the proprioceptive cues to movement (changes in joint angles etc.) were altered on the wall, or perhaps effort is a factor in determining distance traveled (although a priori this might have been expected to compress, rather than expand, the grids).

### Altered Place Cell Firing on the Wall.

As with the grid cells, there are several possible reasons for the attenuated place cell firing that we saw on the wall, including disrupted locomotor inputs, a reduced speed signal, or altered vestibular drive. Indeed, it is possible that on the wall, place cells are relatively deprived of self-motion inputs and are forced to rely more on conjunctions of sensory cues: evidence from virtual reality suggests that around 25% of place cells can function using visual cues alone (39). By this view, something about the altered state of the spatial system on the wall causes fewer place fields to fire on the wall, and this, in turn, reduces drive onto the grid cells and alters the spatial pattern of their firing. However, the effect on place cells, rather than (or as well as) being a direct effect of altered self-motion cues, might instead derive from the grid cells. It is not clear why this would reduce the number but not the size/shape of place fields—however, the transformation from grid cells to place cells is not yet understood generally, and it may be that grid cells supply an excitatory drive to place cells but do not control the spatial properties of place fields.

Putting the above together, it seems likely that the effects of spatial firing on the wall derive from altered processing of linear self-motion cues, but it remains an open question whether these alterations impact grid cells directly or involve a contribution fed back from the place cells.

### Implications of Our Finding for the Structure of the 3D Spatial Map.

Our findings are relevant to the question of how the brain’s spatial systems organize their representation of 3D space. Given recent findings that grid and place cells might express “fields” in a nonspatial dimension (40, 41), this might even have relevance for higher-dimensional conceptual spaces.

A priori, one might have postulated that grid fields would extend into 3D volumetric space in the form of a hexagonal lattice, a vertically elongated lattice, or as stacked grids or as columns of firing (for a theoretical discussion, see refs. 42–44). However, the present results are not compatible with any volumetric model since there is no uniform distribution of firing fields in volumetric space that could simultaneously possess the properties that we saw on each of the two surfaces. For example, firing fields were larger and more spaced apart on the wall than on the floor, and so they cannot both be cross-sections through the same volumetric pattern. The results are, however, consistent with a planar model in which grids are aligned with the locomotor surface and extend orthogonally to this surface, becoming columnar in a volumetric space. In support of a planar model, place cells follow a tilted surface in both normal gravity and microgravity (45, 46), and grid fields also follow a tilted surface (10), while head direction cells transfer their horizontal firing patterns to a wall (33, 34).

Alternatively, there may be two 3D encoding schemes: one for surface travel and one for volumetric travel. Investigations of volumetric spatial exploration in flying bats support this idea: in this situation, place fields are isotropic (8) and head direction cells show sensitivity to all three dimensions (7). These findings raise the possibility that in a volume, the representation becomes isotropic, although the differences seen experimentally may derive from species rather than environment differences. However, if the metric encoding of space depends on the regular packing of grid fields, then theoretical considerations suggest that isotropic encoding is in either species unlikely, because the properties of a 3D hexagonal close-packed lattice are not in themselves isotropic (43). In any case, emerging results suggest that the grid cell pattern in three dimensions does not remain regular, at least in bats.* To the extent that the grid metric underlies the metric encoding of space (which admittedly is still an open question), it thus seems unlikely that vertical and horizontal space are encoded with the same resolution.

In summary, then, we find that the spatial firing patterns of grid and place cells are altered on a vertical plane, with alterations in the spatial pattern of grid fields and the number of firing fields of place cells. Overall, our results suggest that the 3D metrics of the cognitive map are determined by an interaction between egocentric information (the body plane) and allocentric information (the gravity axis). As well as shedding light on the processes underlying grid cell odometry, they point to the importance of gravity in organizing the encoding of 3D space and suggest that the brain’s conception of 3D space, if indeed it has a single conception of it, is inherently anisotropic. If so, this may mean that the subjective perception of spatial scale differs for vertical vs. horizontal travel.

## Methods

Subjects were adult male Lister Hooded rats raised in enriched 3D housing (*SI Appendix*, *SI Text*). All procedures were approved by the University College London Animal Welfare and Ethical Review Body and licensed by the UK Home Office following the revised Animals (Scientific Procedures) Act regulations (2013) modified by the European Directive 2010/63/EU (https://assets.publishing.service.gov.uk/government/uploads/system/uploads/attachment_data/file/619140/ConsolidatedASPA1Jan2013.pdf). Recordings were made from tetrodes implanted in the mEC or HPC, as rats explored either an open field arena, or the “Floor-Wall apparatus” (Fig. 1*A*), which comprised two rectangular (120 × 80 or 120 cm) plywood surfaces, one horizontal and one vertical, adjoining at their short walls. The entire perimeter of the two-surface apparatus was surrounded with 80-cm walls. Both floor and wall were covered with chicken wire. Horizontal battens on the wall, 1 cm thick and 2 cm wide, served to hold the wire away from the wall to allow the animals to cling to it while climbing. Two cameras, one facing the wall and one looking down on the floor, captured the position of a head-mounted LED on the rat’s head as it explored. Spike data were captured and extracted as described in *SI Appendix*, *SI Text* to determine spatial firing patterns, firing rate/speed correlations and rhythmicity. Local field potential data were filtered for theta band activity and analyzed for frequency, power, and phase.

## Supplementary Material

Supplementary File

## Footnotes

*Ginosar G, Finkelstein A, Rubin A, Las L, Ulanovsky N (2015) 3D grid cells and border cells in flying bats, Society for Neuroscience 2015 Meeting, October 17–21, 2015, Chicago.
