# Supplementary material for: Altered neural odometry in the vertical dimension
Source: Proc Natl Acad Sci U S A. 2019 Feb 15;116(10):4631–6. doi: 10.1073/pnas.1811867116 (PMC6410878; doi:10.1073/pnas.1811867116)
Supplement: Supplementary File [file pnas.1811867116.sapp.pdf]

PNAS
www.pnas.org

Supplementary Information for

Altered neural odometry in the vertical dimension

Giulio Casali, Daniel Bush, Kate Jeffery

Email: k.jeffery@ucl.ac.uk

This PDF file includes:

Supplementary text
Figs. S1 to S9
Caption for movie S1
References for SI reference citations

Other supplementary materials for this manuscript include the following:

Movie S1

www.pnas.org/cgi/doi/10.1073/pnas.1811867116

Supplementary Information

Materials and methods

Subjects. Subjects were 14 adult male Lister Hooded rats (Charles-River, UK) weighing ~300g at the start of the experiment. After one week of habituation in standard cages, animals were moved to a large parrot cage with dimensions of 2.6 x 1.6 x 2.2 m (width x depth x height) which was filled with climbing apparatus (ropes, bridges etc.) to familiarize the animals with exploring three-dimensional space (see Supplementary video). Partial light-cycle shift (6h) allowed recordings during their circadian dark cycle. Post-operatively, the animals were mildly food restricted to 90% free-feeding weight and were weighed and checked daily**.** All procedures were licensed by the UK Home Office following the revised ASPA regulations (2013) modified by the European Directive 2010/63/EU.

Electrodes and surgery

Recordings were made using bundles of 4 or 8 tetrodes, each composed of four twisted 25μm or eight twisted 17μm polyimide-coated platinum-iridium (90%/10%) wires (California Fine Wire, CA), attached to an Axona microdrive (Axona Ltd, Herts, UK). Three rats underwent tetrode implantation aimed at the left hippocampus (co-ordinates in mm from bregma: AP: -4.0, ML: 2.5, DV: 1.0) and 11 aimed at the left mEC (co-ordinates in mm from lamda: AP: 1.2, ML: 4.5, DV: 1.5). After the surgery, rats received meloxicam mixed with condensed milk for three days as post-operative analgesia, and were given at least 7 days to recover before the experiment began.

Apparatus

Screening took place in an open-field arena (100 x 100 cm or 120 x 120 cm) surrounded by 80 cm high walls and located in a standard laboratory room. The apparatus was plywood, painted black and polarized by a white cue card above one wall. Most experimental recordings took place on the Floor-Wall apparatus (Fig. 1A), which comprised two rectangular (120 x 80 or 120 cm) plywood surfaces, one horizontal and one vertical, adjoining at their short walls. The entire perimeter of the two-surface apparatus was surrounded with 80 cm walls. Both floor and wall were covered with chicken wire. Horizontal battens on the wall, 1 cm thick and 2 cm wide, served to hold the wire away from the wall to allow the animals to cling to it while climbing. Two cameras, one facing the wall and one looking down on the floor, captured the position of a head-mounted LED on the rat's head as it explored.

Recording setup and procedure

On recording days, each rat was brought into the screening room and connected to the recording apparatus (DacqUSB, Axona Ltd, St Albans, UK) via a lightweight cable connecting the headstage to the preamplifier. To extract unit activity, the signal from the electrodes was amplified 8000-38000 times, bandpass filtered between 500 Hz and 7 kHz and sampled at 50 kHz. To determine local field potential (LFP) activity, the signal from

one of the electrodes was sampled at 250 Hz and bandpass filtered between 0.33 and 125 Hz. The position of the animal was detected and synchronized with the neural signal using an infra-red camera (50 Hz sampling) tracking one or two small single LEDs placed on the animal's head-stage. When place or grid cells were identified, the animals were moved into the recording room, connected to a similar recording system and allowed to forage freely over both surfaces of the floor-wall apparatus for food reward (malt soft paste extra, Gimcat), until sufficient coverage of both surfaces had been attained (2-3 h). In order to link with the recording system, the images from the two cameras were merged into a single image by interleaving their frames with a timebase corrector. The images were then de-interleaved offline, a procedure which reduced sampling frequency to 25 Hz. At the end of each recording day, tetrodes were moved 50-100 µm to reduce the likelihood of the same cells being recorded on consecutive days.

Data analysis

Spike sorting was performed using the automated clustering algorithm Klustakwik 3.0 (1) followed by manual refinement using the graphical cluster-cutting software Tint (Axona Ltd). The path of the animal was reconstructed by smoothing the tracker position points with a time window of 400 ms and interpolating missing points.

Spatial analysis

A spatial rate map for each cell was produced by binning the environment into 2 x 2 cm spatial bins and dividing the number of spikes emitted in that region by the total time the rat spent there; the resulting map was then smoothed with a Gaussian kernel of standard deviation = 4 cm.

Individual firing fields of place and grid cells were extracted as described in Hayman et al. 2015 (2). First the local maxima were determined, and then for each maximum, that bin plus all contiguous bins down to a defined threshold of 35% of that peak were assigned to that field. Firing fields were accepted into further analysis if they exceeded a minimum size of 60 cm^2, a minimum peak rate of 1 Hz and a minimum number of spikes (1% of the total spikes over that recording surface for grid cells and 10% for place cells). The fields then underwent analysis for peak rate, size, long and short axes, and orientation (angle of the long axis). Coverage was determined as the sum of the bins forming the firing fields divided by the sum of the overall bins. Coherence was determined as the correlation between the smoothed vs unsmoothed rate maps, and stability was determined as the correlation between rate maps from the first vs second half of the recording session. Spatial information (3) was obtained using the following formula:

$$I(R|X) \approx \sum_i p(x_i)\ f(x_i)\ log_2\left(\frac{f(x_i)}{F}\right)$$

where $p(x_i)$ and $f(x_i)$ correspond to the probability of the animal being in location (spatial bin) x_i and the firing rate of cell in location x_i, respectively. F is the overall firing

rate and $I(R|X)$ corresponds to the amount of spatial information between firing rate R and location X. The spatial information in bits/spike was calculated by dividing the $I(R|X)$ by F.

Spatial autocorrelograms were used to assess field size, asymmetry score, and grid score (4). They were determined from the smoothed ratemaps using the following formula:

$$r(\tau_x, \tau_y) = \frac{n\Sigma\lambda(x,y)\lambda(x-\tau_x, y-\tau_y) - \Sigma\lambda(x,y)\lambda(x-\tau_x, y-\tau_y)}{\sqrt{n\,\Sigma\lambda(x,y)^2 - (\Sigma\lambda(x,y))^2}\sqrt{n\Sigma\lambda(x-\tau_x, y-\tau_y)^2 - (\Sigma\lambda(x-\tau_x, y-\tau_y))^2}}$$

where $r(\tau_x, \tau_y)$ is the correlation coefficient between those bins with spatial offset τ_x and τ_y, $\lambda(x, y)$ is the firing rate in the spatial bin defined by coordinate x and y, and n is the total number of spatial bins. Field size is the sum of all bins in the central autocorrelation peak exceeding a value of 0.2. The asymmetry score is the normalized aspect ratio ($\frac{a-b}{a+b}$) of the vertical to horizontal extent of the central peak. To account for grid ellipticity, the resulting spatial autocorrelation underwent ellipticity correction similar to that used in Brandon et. al (2011) prior to grid score quantification (5). Firstly, an ellipse was fit through the 6 closest peaks to the center of the spatial autocorrelogram, so that the major and minor axes of the ellipse could be obtained. Secondly, the spatial autocorrelogram was rotated so that the major axis of the fitted ellipse was parallel to the horizontal axis. Thirdly, the spatial autocorrelogram was increased along the y-axis with a 2D linear interpolation so that the minor axis of the ellipse matched the major axis. The resulting spatial autocorrelogram was then rotated back to the original orientation of the major axis of the ellipse and cropped to maintain the original size of the spatial autocorrelogram. Finally, a rotational autocorrelation conducted on the central and 6 surrounding peaks of the autocorrelogram was used to determine grid score, this being the difference between the lowest of the expected peaks (at 60° and 120°) and the highest of the expected troughs (at 30°, 90° and 150°). These calculations were repeated in each iteration of both observed data in the bootstrapping procedures and shuffle data (see Cell acceptance criteria below).

Speed analysis

The speed modulation of each cell was analyzed in two ways. First, we calculated the Pearson correlation between the instantaneous speed of the animal and the instantaneous firing rates smoothed with a Gaussian kernel (260ms window). Second, we corrrelated the firing rate of the cell as a function of running speed between 2 and 20 cm/s, in bins of 2 cm/s (6). A linear regression of the resulting points yielded the slope and intercept of the rate/speed line.

Temporal analysis

Local field potential (LFP) epochs that occurred during running speeds of 2-20 cm/s were analyzed in order to determine the frequency and amplitude of theta rhythmicity, as well as the theta phase of neuronal spiking.
First, power spectral density was obtained using the fast Fourier transform and smoothed with a Gaussian kernel of standard deviation = 0.375 Hz (7). Theta power was then quantified as the ratio between power in a ± 1 Hz window around the peak in the 7-11 Hz range and mean power across the 0-125 Hz range.

The linear relationship between speed and theta frequency was then determined for each session across all surfaces. First, LFP was filtered in the 7-11Hz range using a second order Butterworth filter, and the Hilbert transform used to determine instantaneous phase and amplitude of LFP theta oscillations. Instantaneous LFP theta frequency was estimated from the change in phase between samples, down sampled from 250 to 50 Hz by averaging 5 consecutive samples per bin.

The consistency of the speed-theta relationship was obtained by correlating running speed (again, in 2 cm/s bins across the 2-20 cm/s range) with mean LFP theta frequency in the corresponding speed bins. In addition, linear regression of running speed vs LFP theta frequency provided values for both the intercept, defined as the LFP theta frequency at speed = 0 cm/s, in Hz; and slope, defined as the gradient of the relationship, in Hz/cm/s.

Spike train theta power and frequency, as well as the relationship between these parameters and running speed, were computed using maximum likelihood estimation methods as described in Climer et al. 2015 (8). Briefly, this is achieved by computing the log likelihood that the spike train temporal autocorrelation (restricted to lags of 0.6s, and to periods during which running speed lay in the 2-20 cm/s range) is generated by a Poisson process with a rhythmic rate function and exponential decay. The specific oscillatory amplitude and frequency in the 7-11Hz theta range that produces the highest log likelihood value provides the spike train theta power and frequency estimate for that cell. Significance is computed by comparing this log likelihood value to that generated by a Poisson process with exponential decay only, but no rhythmicity. Finally, the intercept and slope of the running speed/theta frequency relationship can be estimated by generalizing this method to a series of temporal auto-correlations generated across a range of different running speeds.

For both grid and speed cells, the strength of theta phase-locking was also determined by computing the Rayleigh vector length of the relationship between firing rate and co-recorded LFP theta phase, extracted using the Hilbert transform as described above, in the 2-20 cm/s speed window.

Cell acceptance criteria

To avoid regression-to-mean effects we applied the selection criteria in an unbiased way to all three surfaces (floor, wall and open field). Similar to methods used in Savelli et al (2017), we used a bootstrapping approach (100 iterations) to determine the spatial coding of each individual cell by resampling the original spike-train using a bootstrap with replacement procedure (9). In each iteration all spatial parameters (grid score, spatial information, speed score etc) were obtained on each surface. An entorhinal cell was classed as a grid cell if, on at least one of the three experimental surfaces, it met all of the following criteria: a) median grid score obtained from bootstrapping exceeding the 95th percentile of a shuffled distribution obtained by circularly shifting (200 times) the spike train of the same cell by a random delay > 30 seconds; b) mean firing rate < 3 Hz; c) peak firing rate > 1 Hz; d) number of detected fields ≥ 1. A hippocampal cell was classed as a place cell if on at least one of the three experimental surfaces it met all the following criteria: a) median spatial information obtained from bootstrapping ≥ 0.5 bits/spike; b) mean firing rate between 0.1 Hz and 5 Hz; c) peak firing rate > 1 Hz; d) number of detected fields ≥ 1. A cell was classed as a speed cell if on at least one of the three experimental surfaces both the median speed score and speed line score obtained from bootstrapping exceeding the 95th percentile of a shuffled distribution obtained by circularly shifting (200 times) the spike train of the same cell by a random delay > 30 seconds.

Statistics

Values reported are mean +/- standard error unless otherwise stated. Tests were two-tailed unless otherwise stated. A significance level of $p < 0.05$ was used throughout. The Matlab codes for the raincloud-plots visualizations were developed from Allen et al. (2018) (10).

Histology

At the end of the experiment rats were deeply anesthetized, perfused with saline followed by a 4% paraformaldehyde solution (PFA), and the brains removed and stored in solution of 4% PFA. 24 h prior to sectioning, the brains were placed in 20% sucrose and then sections (40 µm thick) were cut on a freezing microtome either sagittally (mEC implanted rats) or coronally (HPC implanted rats), mounted onto Superfrost Plus slides (Thermo Scientific) and stained with cresyl violet or thionin. The site of recording was ascertained by pairing visual information from the microscope (Leica DM750) with estimates of the distance that the electrodes were moved during recording.

Data and code availability

The raw and analyzed data and code that support the findings of this study are available from https://discover.ukdataservice.ac.uk/series/

Supplementary references

1. Kadir SN, Goodman DFM, Harris KD (2014) High-dimensional cluster analysis with the masked EM algorithm. Neural Comput 26(11):2379–2394.

2. Hayman RMA, Casali G, Wilson JJ, Jeffery KJ (2015) Grid cells on steeply sloping terrain: evidence for planar rather than volumetric encoding. Frontiers in Psychology 6. doi:10.3389/fpsyg.2015.00925.

3. Skaggs WE, McNaughton BL, Gothard KM (1993) An Information-Theoretic Approach to Deciphering the Hippocampal Code. Advances in Neural Information Processing Systems 5, eds Hanson SJ, Cowan JD, Giles CL (Morgan-Kaufmann), San Francisco, California pp 1030–1037.

4. Sargolini F, et al. (2006) Conjunctive representation of position, direction, and velocity in entorhinal cortex. Science 312(5774):758–762.

5. Brandon MP, et al. (2011) Reduction of theta rhythm dissociates grid cell spatial periodicity from directional tuning. Science 332(6029):595–599.

6. Wills T, Barry C, Cacucci F (2012) The abrupt development of adult-like grid cell firing in the medial entorhinal cortex. Front Neural Circuits 6:21.

7. Jeewajee A, Lever C, Burton S, O'Keefe J, Burgess N (2008) Environmental novelty is signaled by reduction of the hippocampal theta frequency. Hippocampus 18(4):340–348.

8. Climer JR, DiTullio R, Newman EL, Hasselmo ME, Eden UT (2015) Examination of rhythmicity of extracellularly recorded neurons in the entorhinal cortex. Hippocampus 25(4):460–473.

9. Savelli F, Luck J, Knierim JJ (2017) Framing of grid cells within and beyond navigation boundaries. eLife 6:e21354.

10. Allen M, Poggiali D, Whitaker K, Marshall TR, Kievit R (2018) Raincloud plots: a multi-platform tool for robust data visualization (PeerJ Inc.) doi:10.7287/peerj.preprints.27137v1.

11. Barry C, Burgess N (2017) To be a grid cell: shuffling procedures for determining "gridness." bioRxiv:230250.

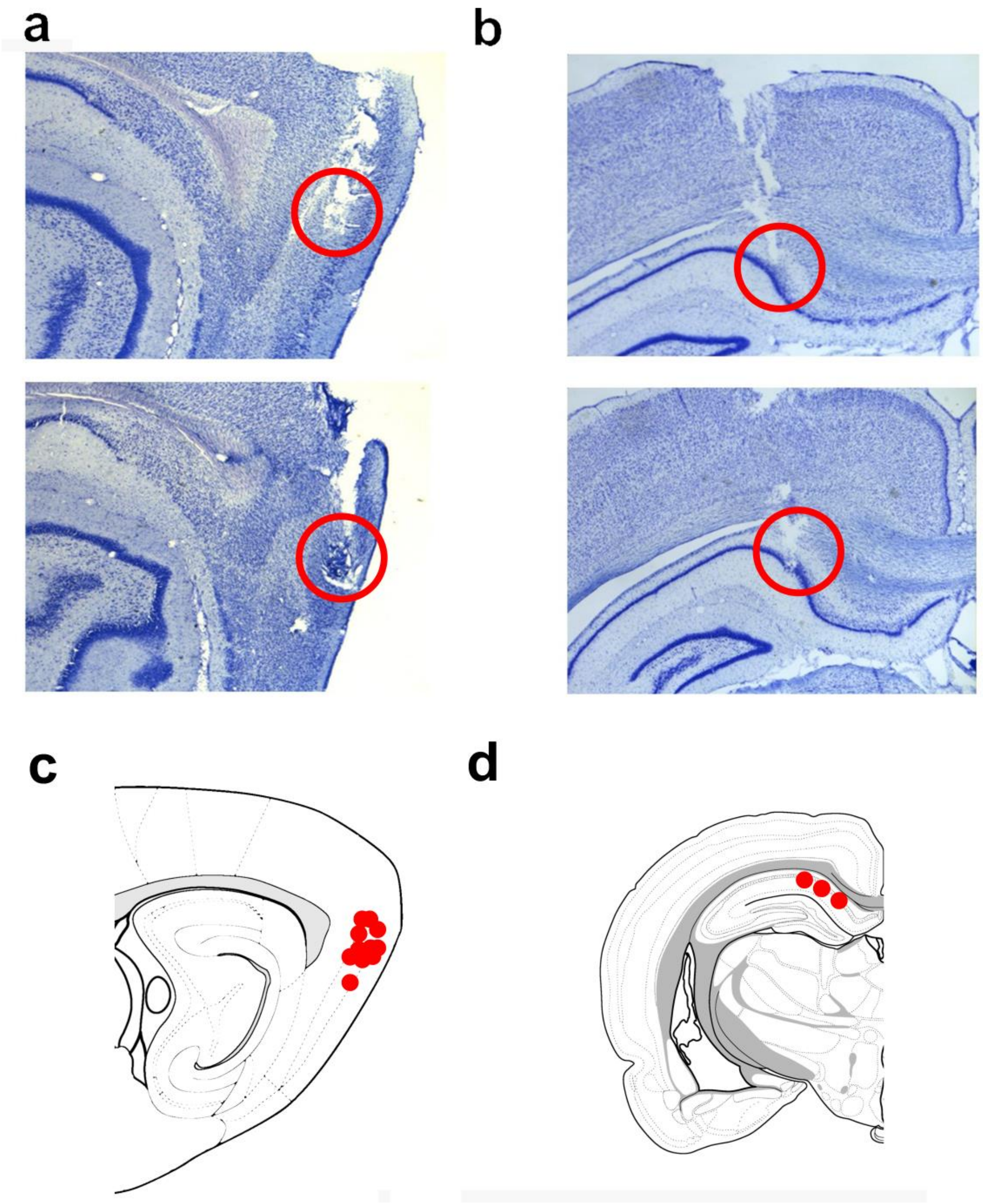

Fig. S1. Histology

(**a-b**) Representative sections of the entorhinal (**a**) and hippocampal (**b**) implantation sites showing electrode tip location (red circles). (**c-d**) Estimated recording sites for the population of entorhinal (**c**) and hippocampal (**d**) implants. Line drawings show a sagittal section at 4.60 mm lateral to midline (**c**), and a coronal section at 4.16 mm posterior to bregma (**d**). Images from Swanson Brain Atlas http://larrywswanson.com/?page_id=164 licensed under a Creative Commons Attribution-NonCommercial 4.0 International License.

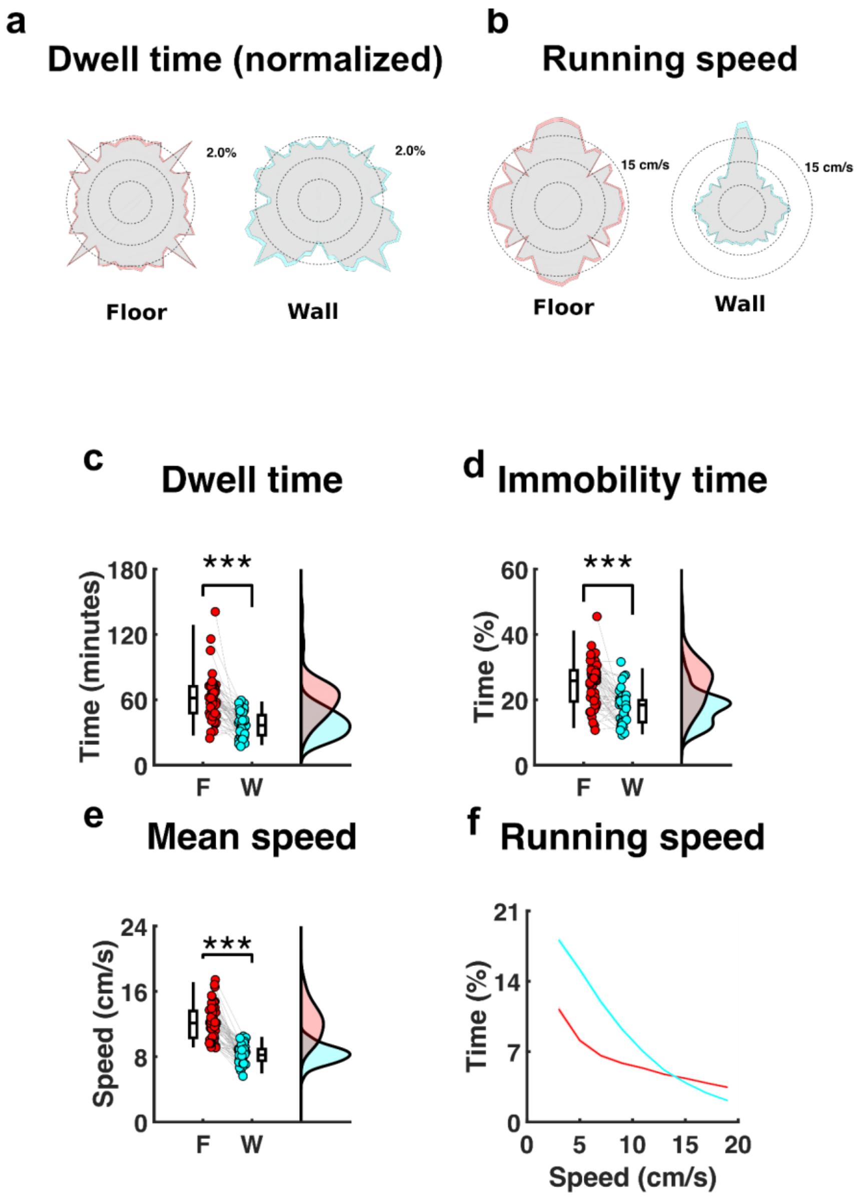

Fig. S2. Behavioural indices

(n = 53 sessions) Polar plots representing mean (solid line) ± s.e.m (colored shading) of the normalized dwell time (**a**) and running speed (**b**) as a function of heading direction and surface. Speed of movement on the two surfaces. The dot plots show individual data together with the mean (solid line) ± s.e.m. (colored shading). Rats spent significantly more time on the floor (**c**, dwell time: floor = 62 ± 21 minutes, wall = 37 ± 11 minutes; t_{48}= 7.73, *P* = 5.64e-10), stayed immobile for relatively more time (**d**, immobility: floor = 25 ± 7 %, wall = 17 ± 5 %; t(48)= 7.53, *P* = 1.15e-09), and showed greater mean speed (**e**, mean speed: floor = 12.2 ± 2.1 cm/s, wall = 8.3 ± 1.0 cm/s; t(48) = 13.0, *P* = 2.62e-17). **f**, The line graph shows the distribution of time spent at different running speeds, as a function of surface (Two-sample Kolmogorov-Smirnov test, stat = 0.16, *P* < 0.0001).

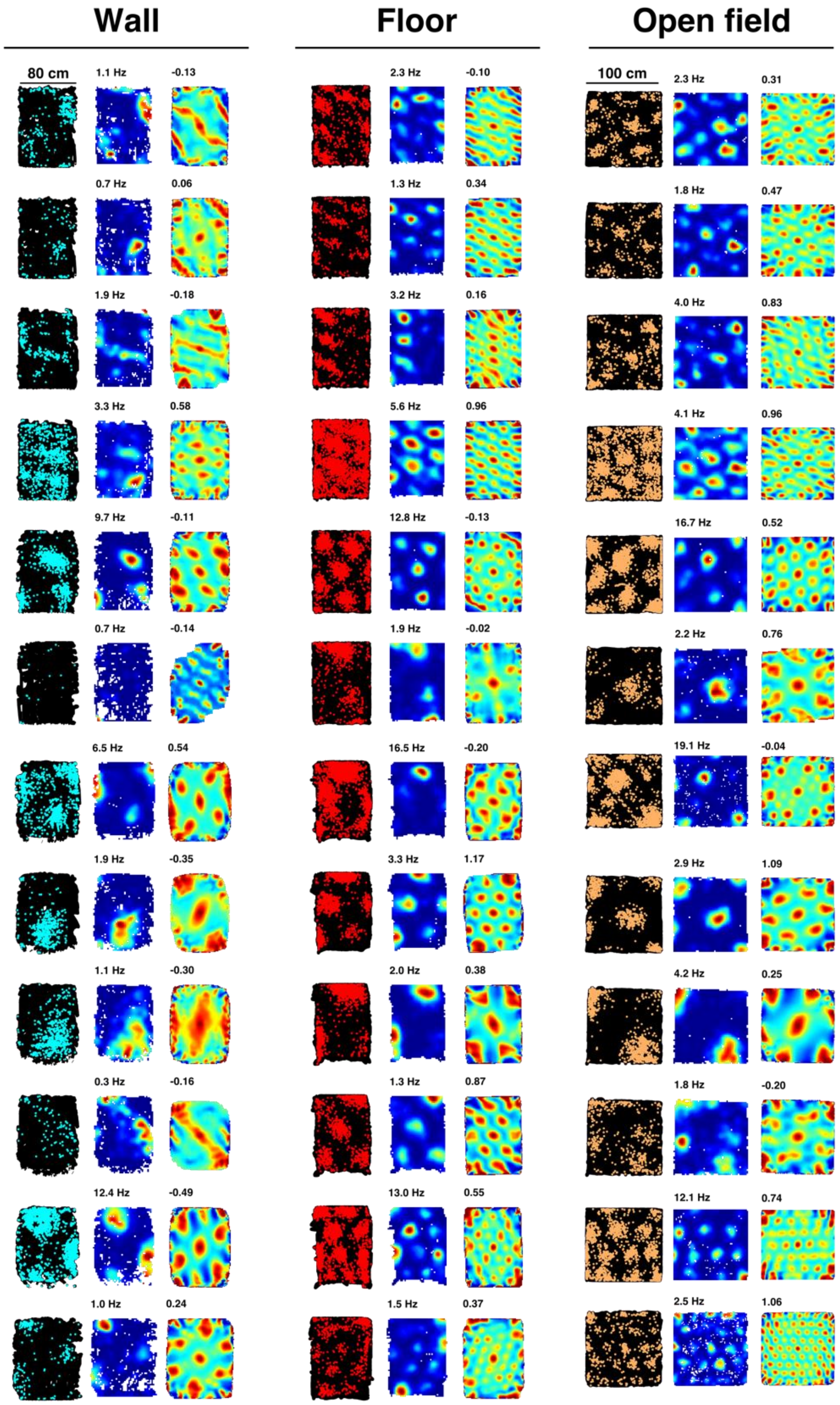

Fig. S3. Spike plots, ratemaps and autocorrelogram of 48 example grid cells recorded across all three surfaces.

Corresponding peak firing rate and grid score are displayed in the top left corner of each ratemap and autocorrelogram, respectively.

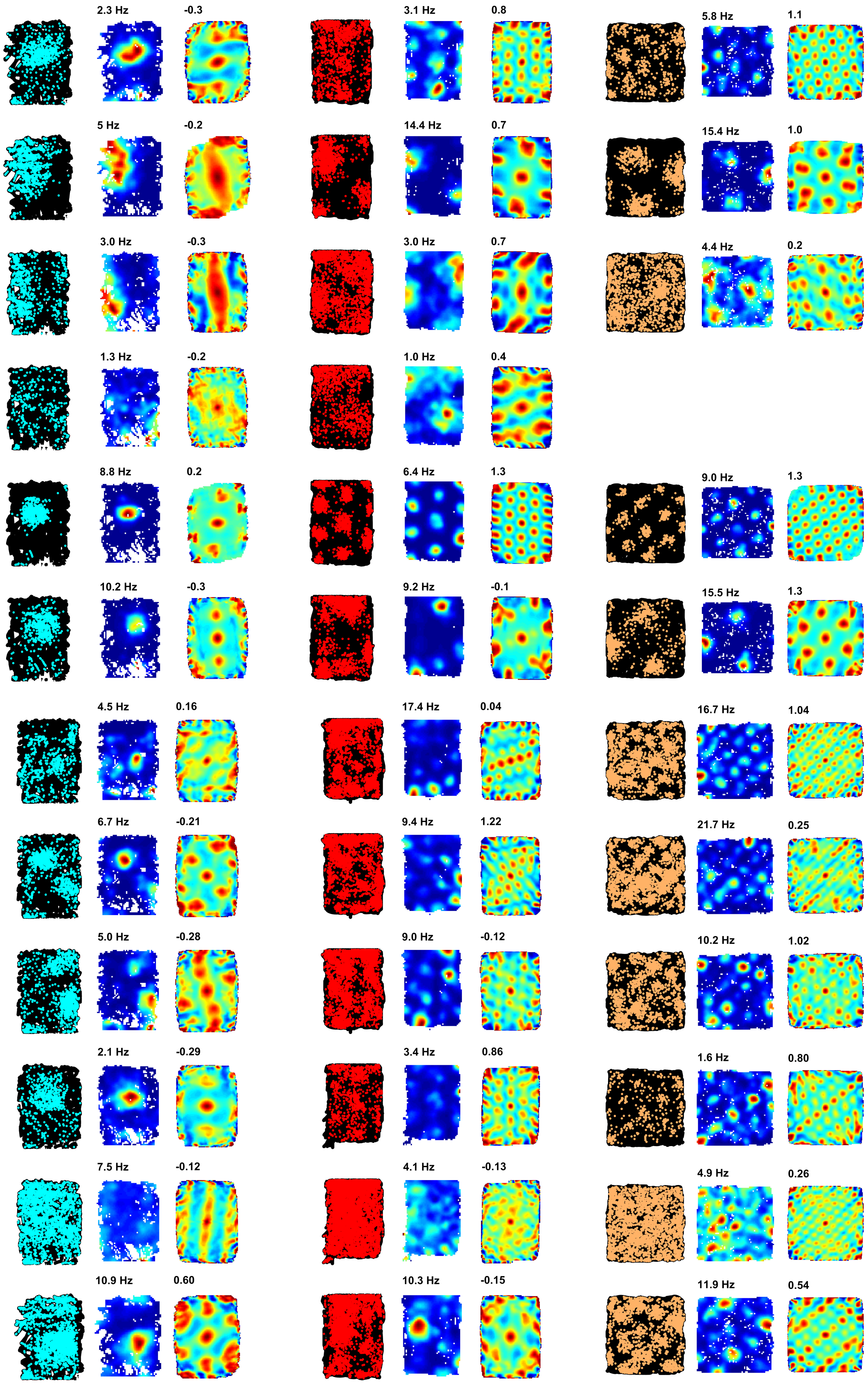

2.3 Hz
-0.3
3.1 Hz
0.8
5.8 Hz
1.1
5 Hz
-0.2
14.4 Hz
0.7
15.4 Hz
1.0
3.0 Hz
-0.3
3.0 Hz
0.7
4.4 Hz
0.2
1.3 Hz
-0.2
1.0 Hz
0.4
8.8 Hz
0.2
6.4 Hz
1.3
9.0 Hz
1.3
10.2 Hz
-0.3
9.2 Hz
-0.1
15.5 Hz
1.3
4.5 Hz
0.16
17.4 Hz
0.04
16.7 Hz
1.04
6.7 Hz
-0.21
9.4 Hz
1.22
21.7 Hz
0.25
5.0 Hz
-0.28
9.0 Hz
-0.12
10.2 Hz
1.02
2.1 Hz
-0.29
3.4 Hz
0.86
1.6 Hz
0.80
7.5 Hz
-0.12
4.1 Hz
-0.13
4.9 Hz
0.26
10.9 Hz
0.60
10.3 Hz
-0.15
11.9 Hz
0.54

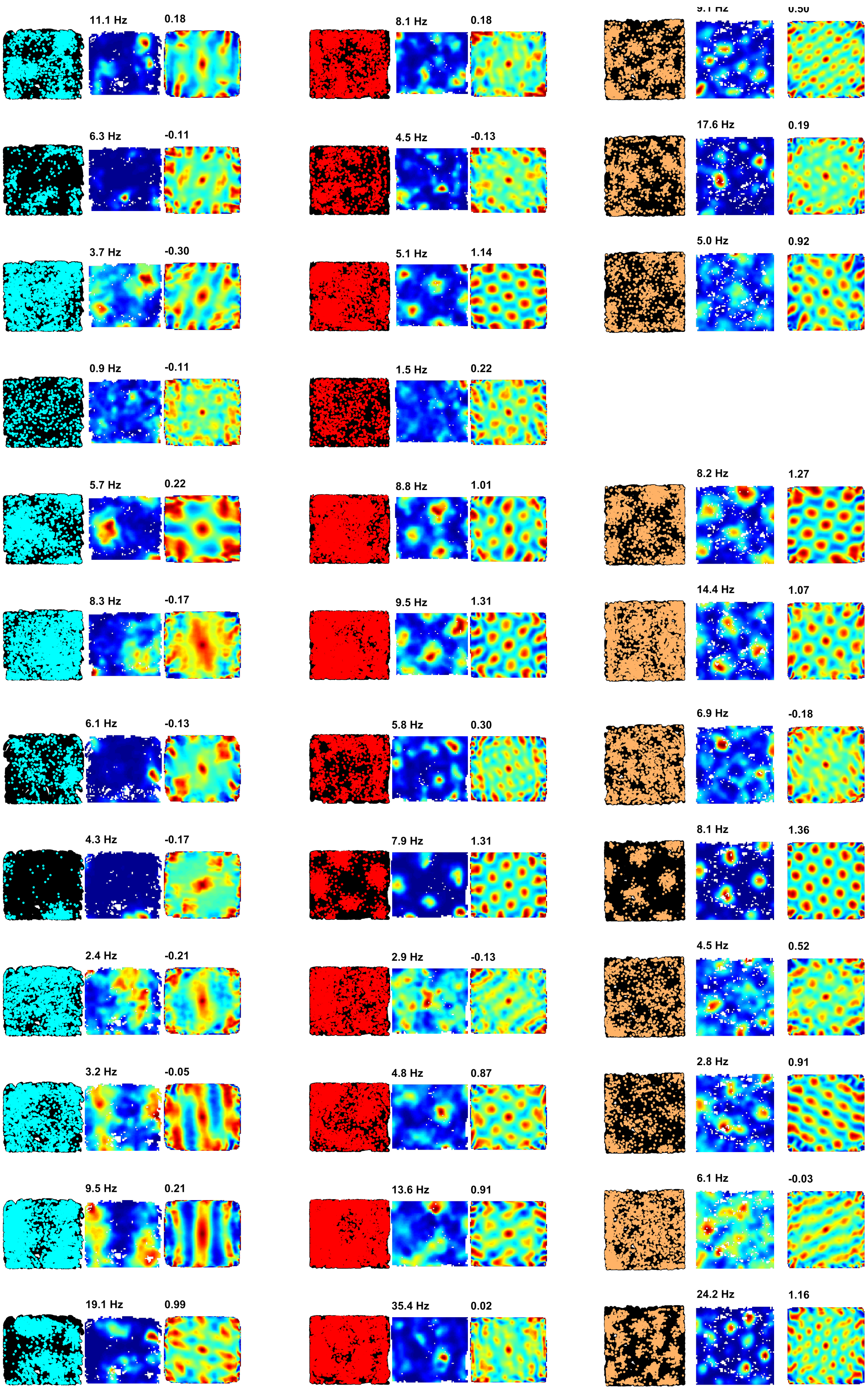
11.1 Hz
0.18
6.3 Hz
-0.11
3.7 Hz
-0.30
0.9 Hz
-0.11
5.7 Hz
0.22
8.3 Hz
-0.17
6.1 Hz
-0.13
4.3 Hz
-0.17
2.4 Hz
-0.21
3.2 Hz
-0.05
9.5 Hz
0.21
19.1 Hz
0.99
8.1 Hz
0.18
4.5 Hz
-0.13
5.1 Hz
1.14
1.5 Hz
0.22
8.8 Hz
1.01
9.5 Hz
1.31
5.8 Hz
0.30
7.9 Hz
1.31
2.9 Hz
-0.13
4.8 Hz
0.87
13.6 Hz
0.91
35.4 Hz
0.02
17.6 Hz
0.19
5.0 Hz
0.92
8.2 Hz
1.27
14.4 Hz
1.07
6.9 Hz
-0.18
8.1 Hz
1.36
4.5 Hz
0.52
2.8 Hz
0.91
6.1 Hz
-0.03
24.2 Hz
1.16

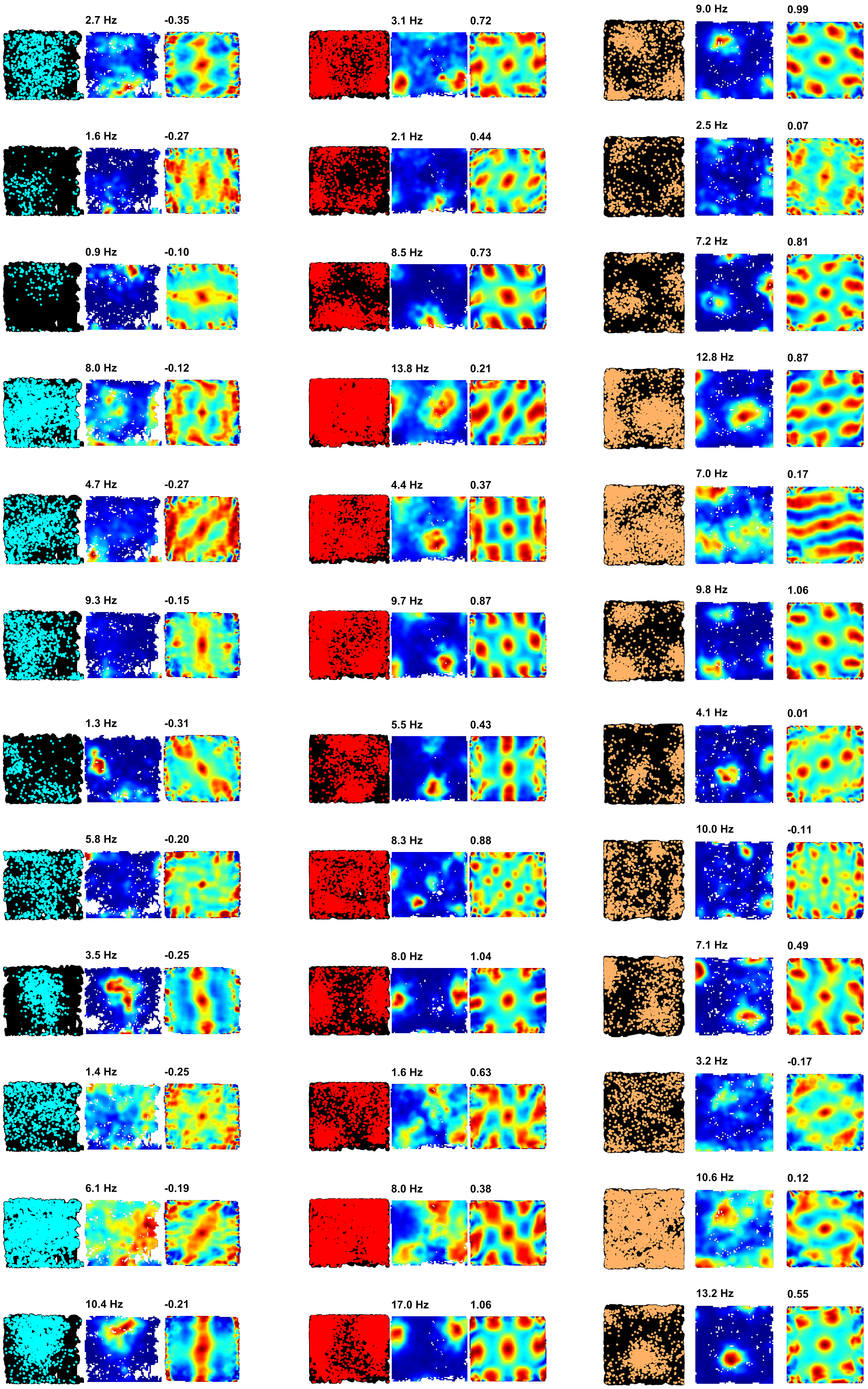
2.7 Hz
-0.35
3.1 Hz
0.72
9.0 Hz
0.99
1.6 Hz
-0.27
2.1 Hz
0.44
2.5 Hz
0.07
0.9 Hz
-0.10
8.5 Hz
0.73
7.2 Hz
0.81
8.0 Hz
-0.12
13.8 Hz
0.21
12.8 Hz
0.87
4.7 Hz
-0.27
4.4 Hz
0.37
7.0 Hz
0.17
9.3 Hz
-0.15
9.7 Hz
0.87
9.8 Hz
1.06
1.3 Hz
-0.31
5.5 Hz
0.43
4.1 Hz
0.01
5.8 Hz
-0.20
8.3 Hz
0.88
10.0 Hz
-0.11
3.5 Hz
-0.25
8.0 Hz
1.04
7.1 Hz
0.49
1.4 Hz
-0.25
1.6 Hz
0.63
3.2 Hz
-0.17
6.1 Hz
-0.19
8.0 Hz
0.38
10.6 Hz
0.12
10.4 Hz
-0.21
17.0 Hz
1.06
13.2 Hz
0.55

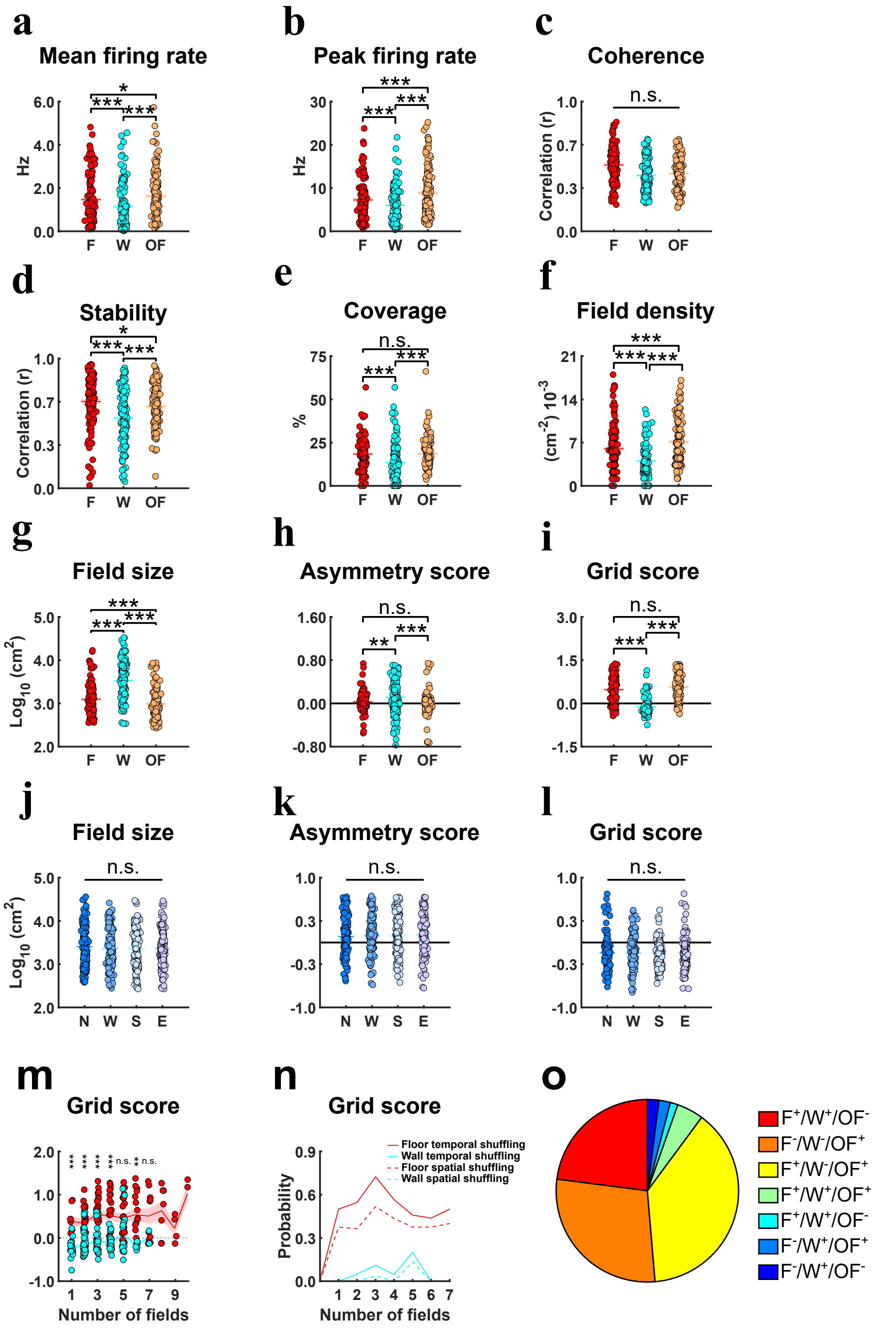

Fig. S4. Grid cell firing indices

139 of the total 148 grid cells were recorded in all three environments. Plots show individual cell values plus mean and s.e.m., * = $P < 0.05$, * * = $P < 0.01$, *** = $P < 0.001$.

Grid cells showed significant differences between surfaces both in the mean firing rate (**a**, floor = 1.5 ± 0.1 Hz, wall = 1.1 ± 0.1 Hz, open field = 1.6 ± 0.1 Hz, $F_{2,276}$ = 33.4, P = 1.05 e^{-13}) and peak firing rate (**b**, floor = 7.2 ± 0.4 Hz, wall = 6.0 ± 0.3 Hz, open field = 8.9 ± 0.5 Hz, $F_{2,276}$ = 34.5, P = 4.00^{-14}). The majority of grid cell spatial properties between surfaces substantially differed between surfaces: the coherence (**c**) was close but not significantly different across surfaces: floor = 0.51 ± 0.01, wall = 0.43 ± 0.01, open

field = 0.44 ± 0.11, $F_{2,276}$ = 2.99, P = 0.052, spatial stability (**d**) was reduced on the wall: floor = 0.67 ± 0.02, wall = 0.54 ± 0.02, open field = 0.63 ± 0.01, $F_{2,274}$ = 1.20, P = 4.01 e^{-10} while coverage (**e**) was increased; floor = 18.4 ± 0.8 %, wall = 13.4 ± 0.9 %, open field = 18.6 ± 0.7 %, $F_{2,276}$ = 21.4, P = 2.32e^{-09}.

Spatial parameters of grid firing fields also were substantially different across surface: the number of fields (normalized by area, called field density) were reduced on the wall (**f**): floor = 6.02 ± 0.31 x 10^{-3} cm^{-2}, wall = 4.04 ± 0.22 x 10^{-3} cm^{-2}, open field = 7.12 ± 0.30 x 10^{-3} cm^{-2}, $F_{2,276}$ = 60.5, P = 3.50e^{-18}, the size of the fields was increased on the wall (**g**): floor = 3.09 ± 0.03 $\log_{10}$ cm^2, wall = 3.52 ± 0.04 $\log_{10}$ cm^2, open field = 3.00 ± 0.03 $\log_{10}$ cm^2, $F_{2,276}$ = 147.9, P = 1.45e^{-18}, vertical expansion was increased on the wall (**f**), asymmetry score: floor = 0.03 ± 0.01, wall = 0.11 ± 0.03, open field = 0.00 ± 0.02, $F_{2,276}$ = 8.85, P = 7.40 e^{-05}, and the hexagonal regularity dramatically reduced on the wall (**i**)**:** grid score: floor = 0.48 ± 0.04, wall = - 0.11 ± 0.02, open field = 0.57 ± 0.04, $F_{2,276}$ = 125.3, P = 3.28e^{-18}. The field size, asymmetry score and grid score on the wall were also compared across cardinal heading directions (North, West, South, East) and they all showed no significant differences: size of the fields (**j**): $F_{3,441}$ = 1.54, P = 0.20; asymmetry score (**k**): $F_{3,441}$ = 0.49, P = 0.69; gridness (**l**): grid score: $F_{3,441}$ = 0.061, P = 0.98.

m, To test whether the reduced grid score of cells was due to the concomitant reduction in the number of fields, the grid scores of floor and wall between cells with identical number of fields was compared with unpaired t-test with Bonferroni correction (alpha = 0.0045). Our analyses revealed significant decrease on the wall between cells with only 1 field (floor = 0.38 ± 0.13, wall = -0.23 ± 0.04, unpaired t-test, t_{28} = 6.11, P = 1.36e^{-06}), cells with 2 fields (floor = 0.34 ± 0.08, wall = -0.15 ± 0.04, unpaired t-test, t_{30} = 6.39, P = 2.74e^{-08}), cells with 3 fields (floor = 0.54 ± 0.08, wall = -0.08 ± 0.05, unpaired t-test, t_{54} = 6.47, P = 2.98e^{-08}), cells with 4 fields (floor = 0.51 ± 0.11, wall = -0.10 ± 0.04, unpaired t-test, t_{43} = 5.15, P = 6.12e^{-06}), there were no significant changes between cells with 5 fields (floor = 0.46 ± 0.10, wall = 0.07 ± 0.13, unpaired t-test, t_{37} = 2.42, P = 0.020), significant changes between cells with 6 fields (floor = 0.54 ± 0.13, wall = 0.07 ± 0.04, unpaired t-test, t_{19} = 3.08, P = 0.0061), and changes between cells with 7 fields (floor = 0.51 ± 0.18, wall = 0.02 ± 0.15, unpaired t-test, t_{10} = 1.13, P = 0.029).

n, Probability of grid cells showing grid scores greater than 95th percentile null distribution obtained using temporal (solid line) or spatial (dashed line) shuffling procedure (11) on both surfaces (red = floor, wall = turquoise) across cells with increasing number of fields. To explore whether the overall reduction in the number of significant grid cells on the wall could be due to the concomitant reduction in the number of fields, the probability of cells showing same number of fields between floor and wall had significant grid score based on temporal spike shuffling was compared with the Fisher exact test with Bonferroni correction (alpha = 0.0045). For grid cells with 1 field the results were: floor = 3 vs 8, Wall 0 vs 22, P = 0.0026; for grid cells with 2 fields the results were: floor = 12 vs 10, wall = 2 vs 38, P < 0.0001; for grid cells with 3 fields the results were: floor = 21 vs 8, wall = 3 vs 24, P < 0.0001; for grid cells with 4 fields the results were: floor = 13 vs 10, wall = 1 vs 21, P = 0.0002; for grid cells with 5 fields the

results were: floor = 11 vs 13, wall = 3 vs 12, $P = 0.17$; for grid cells with 6 fields the results were: floor = 7 vs 9, wall = 0 vs 5, $P = 0.12$; for grid cells with 7 fields the results were: floor = 5 vs 5, wall = 0 vs 2, $P = 0.47$. Similar results were obtained when we compared the probability of cells showing same number of fields between floor and wall with significant grid score based on spatial rather than temporal shuffling (11). floor = 3 vs 5, Wall 0 vs 22, $P = 0.014$; for grid cells with 2 fields the results were: floor = 8 vs 14, wall = 0 vs 40, $P < 0.0001$; for grid cells with 3 fields the results were: floor = 15 vs 14, wall = 1 vs 27, $P < 0.0001$; for grid cells with 4 fields the results were: floor = 10 vs 13, wall = 0 vs 22, $P = 0.0002$; for grid cells with 5 fields the results were: floor = 9 vs 15, wall = 2 vs 13, $P = 0.15$; for grid cells with 6 fields the results were: floor = 6 vs 10, wall = 0 vs 5, $P = 0.26$; for grid cells with 7 fields the results were: floor = 4 vs 6, wall = 0 vs 2, $P = 0.51$.

o, Pie chart representing percentages of cells classed as significant grid cells on each surface: $F^+W^- O^-$ = 34 cells, $F^-W^-O^+$ = 42 cells, $F^+W^-O^+$ = 57 cells, $F^+W^+O^+$ = 7 cells, $F^+W^+O^-$ = 2 cells, $F^-W^+O^+$ = 3 cells, $F^-W^+O^-$ = 3 cells.

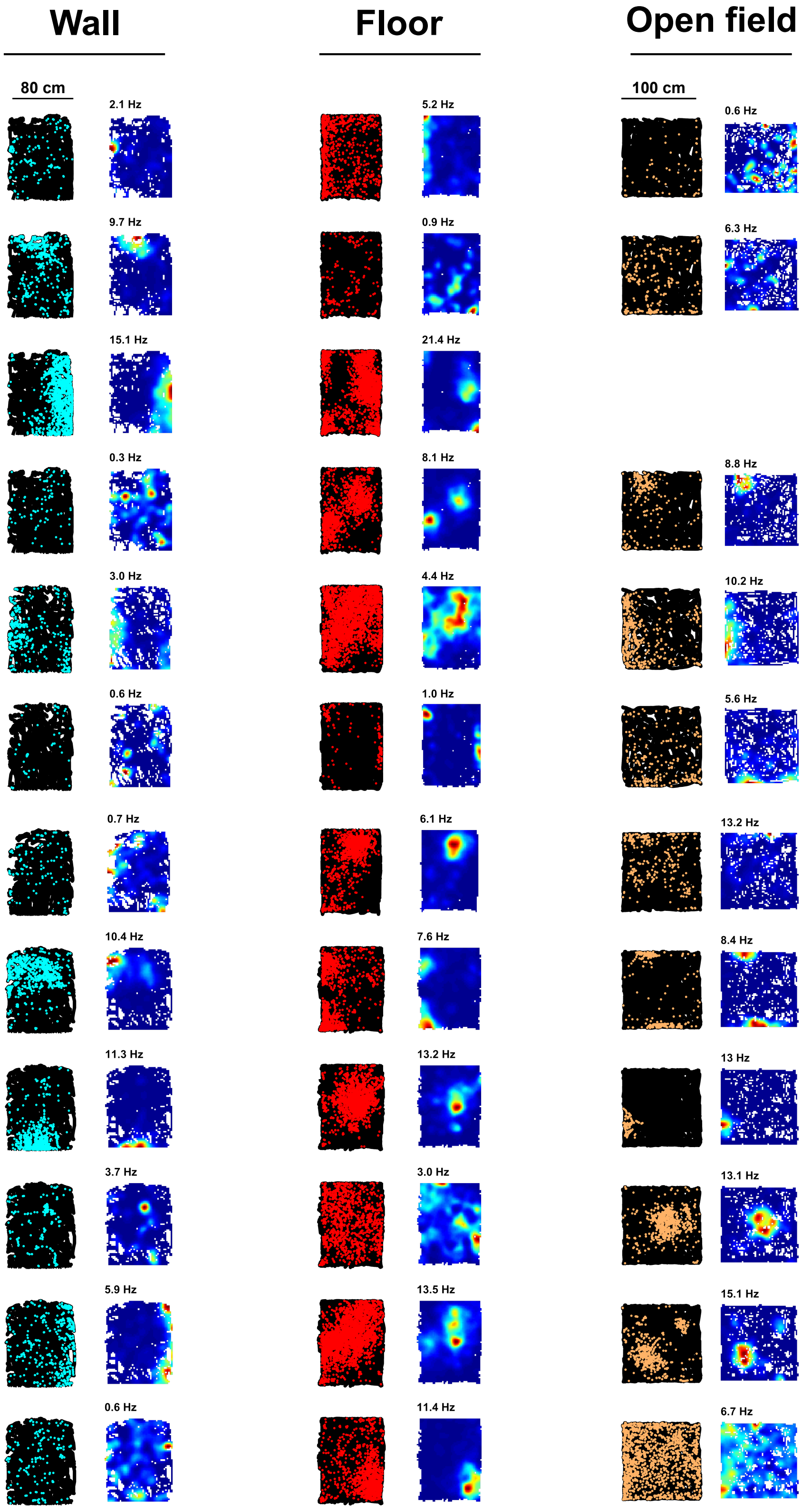

Fig. S5. Spike plots and ratemaps for example place cells
(This page and 3 following). Spike plots and ratemaps of 48 individual place cells recorded across surfaces.

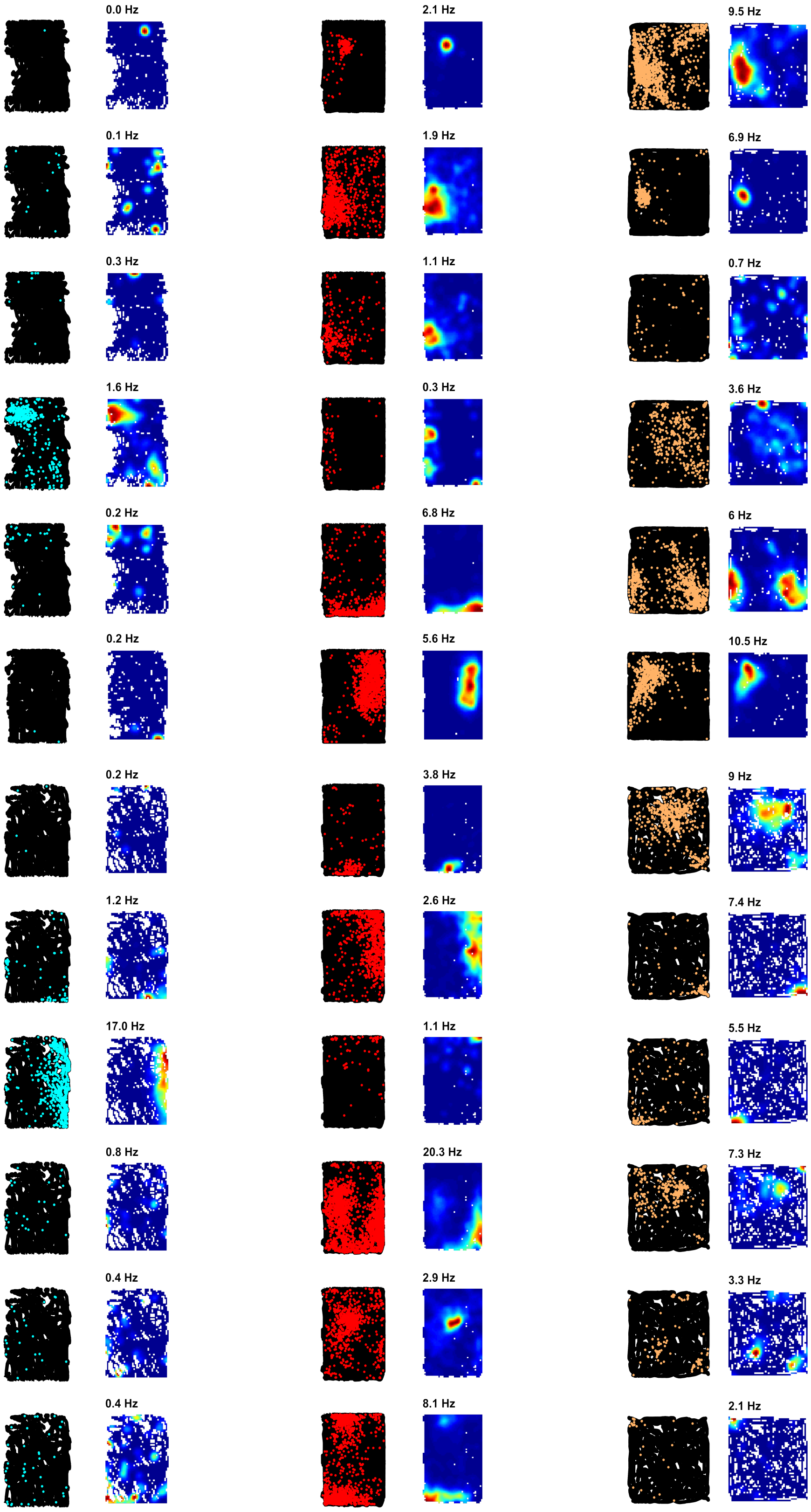
0.0 Hz
2.1 Hz
9.5 Hz
0.1 Hz
1.9 Hz
6.9 Hz
0.3 Hz
1.1 Hz
0.7 Hz
1.6 Hz
0.3 Hz
3.6 Hz
0.2 Hz
6.8 Hz
6 Hz
0.2 Hz
5.6 Hz
10.5 Hz
0.2 Hz
3.8 Hz
9 Hz
1.2 Hz
2.6 Hz
7.4 Hz
17.0 Hz
1.1 Hz
5.5 Hz
0.8 Hz
20.3 Hz
7.3 Hz
0.4 Hz
2.9 Hz
3.3 Hz
0.4 Hz
8.1 Hz
2.1 Hz

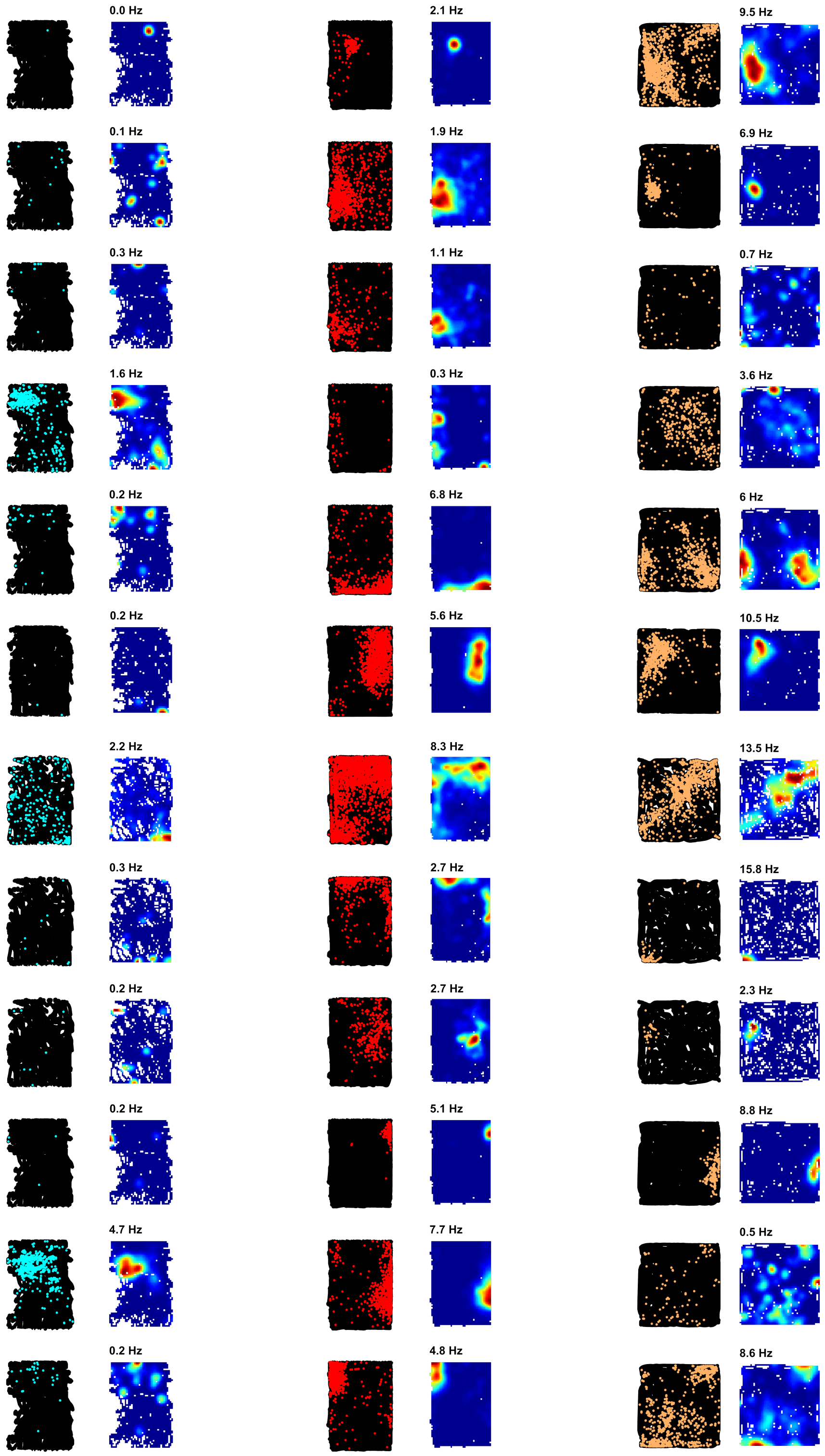
0.0 Hz
2.1 Hz
9.5 Hz
0.1 Hz
1.9 Hz
6.9 Hz
0.3 Hz
1.1 Hz
0.7 Hz
1.6 Hz
0.3 Hz
3.6 Hz
0.2 Hz
6.8 Hz
6 Hz
0.2 Hz
5.6 Hz
10.5 Hz
2.2 Hz
8.3 Hz
13.5 Hz
0.3 Hz
2.7 Hz
15.8 Hz
0.2 Hz
2.7 Hz
2.3 Hz
0.2 Hz
5.1 Hz
8.8 Hz
4.7 Hz
7.7 Hz
0.5 Hz
0.2 Hz
4.8 Hz
8.6 Hz

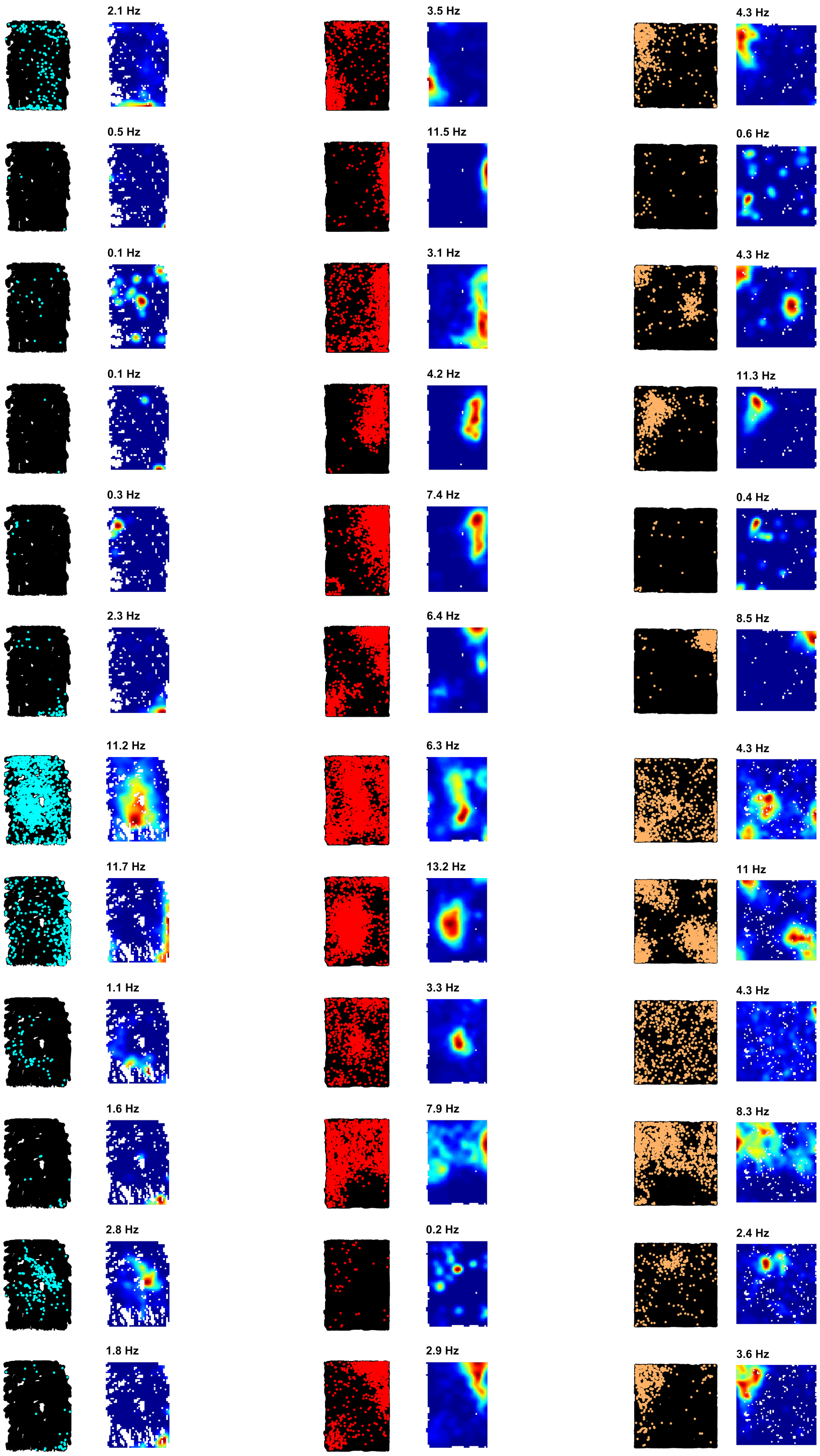
2.1 Hz
3.5 Hz
4.3 Hz
0.5 Hz
11.5 Hz
0.6 Hz
0.1 Hz
3.1 Hz
4.3 Hz
0.1 Hz
4.2 Hz
11.3 Hz
0.3 Hz
7.4 Hz
0.4 Hz
2.3 Hz
6.4 Hz
8.5 Hz
11.2 Hz
6.3 Hz
4.3 Hz
11.7 Hz
13.2 Hz
11 Hz
1.1 Hz
3.3 Hz
4.3 Hz
1.6 Hz
7.9 Hz
8.3 Hz
2.8 Hz
0.2 Hz
2.4 Hz
1.8 Hz
2.9 Hz
3.6 Hz

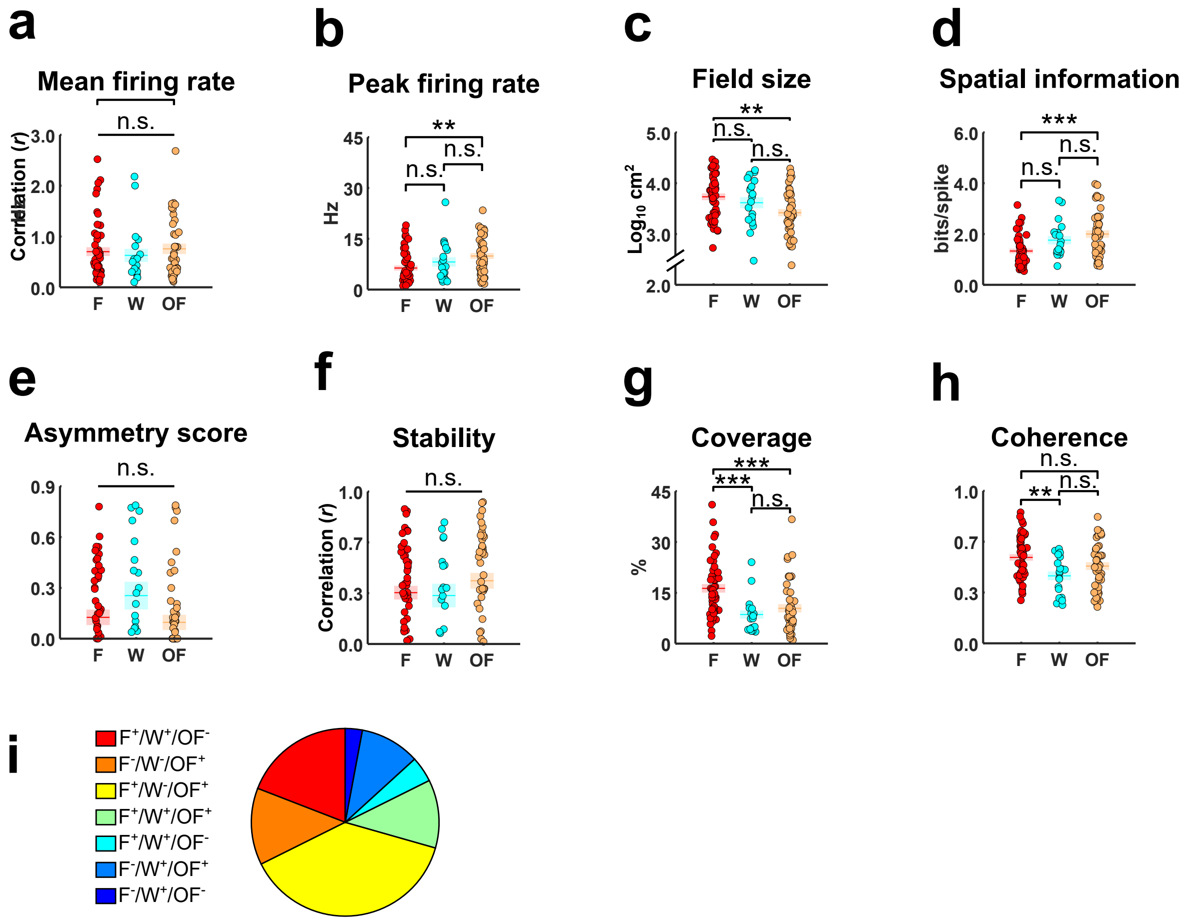

Fig. S6. Place cell firing properties compared between floor, wall and open -field surfaces.

50 of the total 72 place cells were active on the floor, 20 were active on the wall and 49 in the open field. Plots show values of the active cells across surfaces plus mean and s. e. m., * = $P < 0.05$, * * = $P < 0.01$, *** = $P < 0.001$.

Place cells showed no differences between surfaces in the mean firing rate (**a**; floor = 0.70 ± 0.08 Hz, wall = 0.63 ± 0.12 Hz, open field = 0.76 ± 0.10 Hz, $F_{2,112} = 0.32$, P = 0.72) but significant differences in the peak firing rate (**b**; floor = 6.4 ± 0.7 Hz, wall = 8.2 ± 1.3 Hz, open field = 9.9 ± 0.8 Hz, $F_{2,112} = 5.61$, $P = 0.0048$).

The spatial properties of place cell firing differed slightly across surfaces, as follows: size of the fields (**c**; floor = 3.73 ± 0.06 $\log_{10}$ cm^2, wall = 3.61 ± 0.10 $\log_{10}$ cm^2, open field = 3.42 ± 0.07 $\log_{10}$ cm^2, $F_{2,112} = 5.96$, $P = 0.0034$); spatial information (**d**; floor = 1.33 ± 0.08 bits/spike, wall = 1.76 ± 0.15 bits/spike, open field = 2.00 ± 0.14 bits/spike, $F_{2,112} = 9.60$, $P = 0.0001$); asymmetry score (**e**; floor = 0.13 ± 0.04, wall = 0.25 ± 0.08, open field = 0.10 ± 0.04, $F_{2,116} = 1.80$, $P = 0.17$); stability (**f**; floor = 0. 34 ± 0.04, wall = 0.32 ± 0.08, open field = 0.41 ± 0.05, $F_{2,109} = 0.922$, $P = 0.40$); coverage (**g**; floor = 16.3 ± 1.1 %, wall = 8.6 ± 1.1 %, open field = 10.4 ± 1.1 %, $F_{2,112} = 10.5$, $P = 6.83e^{-05}$); coherence (**h**; floor = 0.56 ± 0.02, wall = 0.44 ± 0.03, open field = 0.51 ± 0.02, $F_{2,112} = 5.34$, $P = 0.0061$).

i, Pie chart representing percentages of cells classed as place cells on each surface:

$F^+W^-O^-$ = 8 cells, $F^-W^-O^+$ = 2 cells, $F^+W^-O^+$ = 26 cells, $F^+W^+O^+$ = 13 cells, $F^+W^+O^-$ = 9 cells, $F^-W^+O^+$ = 3 cells, $F^-W^+O^-$ = 7 cells.

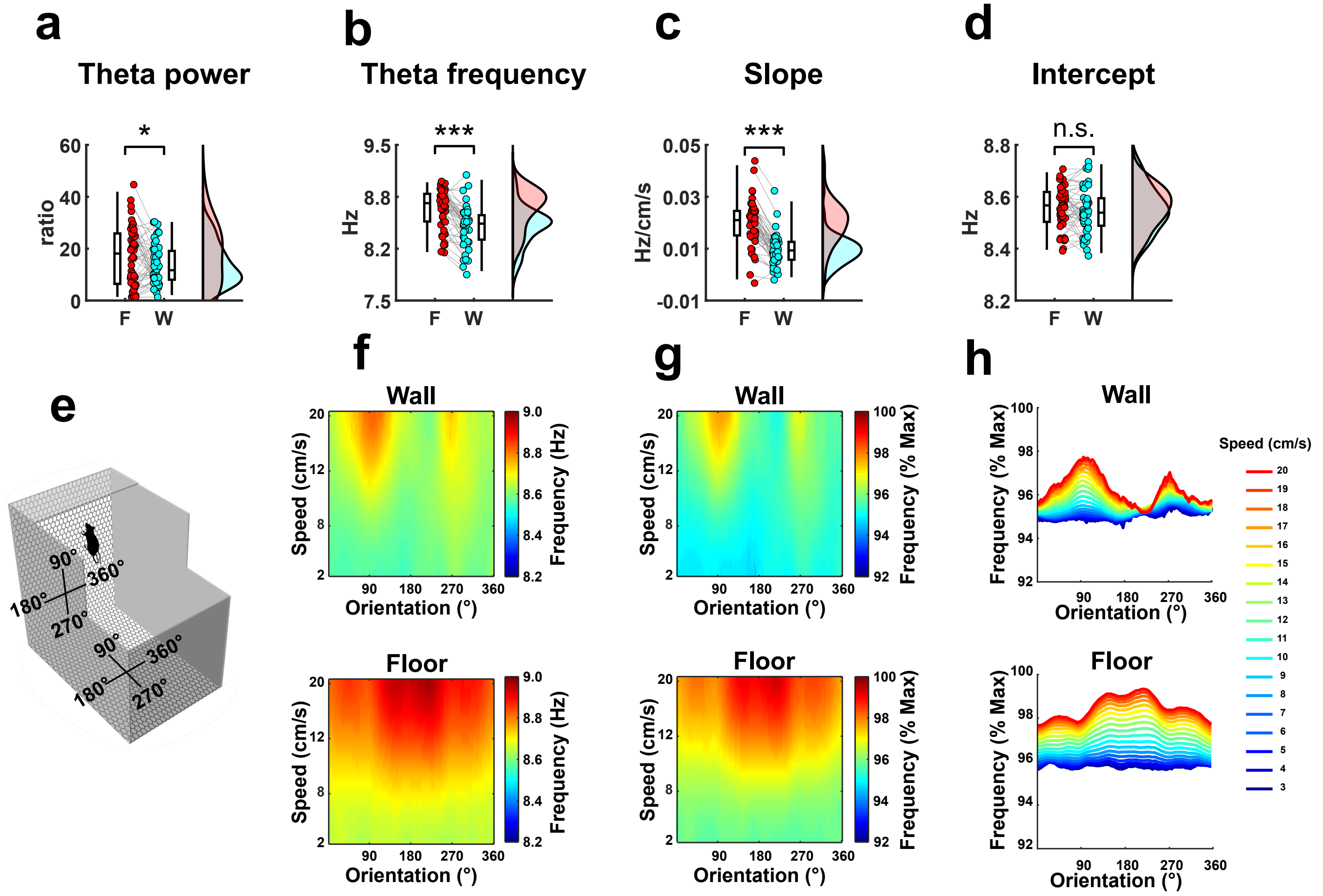

Fig. S7. LFP theta oscillation between floor and wall.
LFP theta oscillation was recorded across 48 sessions between surfaces. Plots show individual session values plus mean and s.e.m., * = $P < 0.05$, ** = $P < 0.01$, *** = $P < 0.001$.

LFP theta showed significant reduced theta power (**a**; peak/mean ratio: floor = 17.1 ± 1.6, wall = 13.7 ± 1.1; $t_{47} = 2.18$, $P = 0.034$) and mean theta frequency (**b**; floor = 8.68 ± 0.04 Hz, wall = 8. 45 ± 0.04 Hz; $t_{47} = 7.13$, $P = 5.20e^{-09}$). Comparisons of the speed-theta frequency relationship revealed a significant reduction in the slope on the wall compared to the floor (**c**; slope: floor = 0.017 ± 0.001 Hz/cm/s, wall = 0.006 ± 0.001 Hz/cm/s; $t_{47} = 10.9$, $P = 2.03e^{-14}$), and no difference in the intercept (**d**; intercept: floor = 8.56 ± 0.01 Hz, wall = 8.55 ± 0.01, $t_{47} = 1.13$, $P = 0.26$).

The observed reduction in LFP theta frequency on the wall prompted to further explore potential effects of heading direction between surfaces. **e**; Schematic representation of the floor-wall apparatus with superimposed heading directions on both surfaces. **f**; Mean LFP theta frequency between surfaces (wall = top, floor = bottom) across heading direction (x-axis) and running speed (y-axis) represented as heat map (blue = 8.2 Hz, red = 9.0 Hz). Note the gradual increase in LFP theta frequency along running speed but not heading direction on the floor. In contrast, LFP theta frequency on the wall was generally reduced and showed increase only along upwards (90°) and downwards (270°) movements. **g**; Similar to **f**, LFP theta frequency between surfaces is shown by means of a 2D heat map (blue = 92 %, red = 100 %), scaled as the percentage of the maximum LFP theta frequency on the floor for each individual session. On the floor there was a gradual

increase along running speed only, whereas on the wall, the increase was only present along vertical movements. **h**; LFP theta frequency shown by lines representing the percentage of the maximum LFP theta frequency on the floor (y-axis) plotted along heading directions (x-axis) for each individual color-coded speed (low speed = dark blue, high speed = red). On the floor, the lines showed little directional modulation, in contrast to the wall where there was a clear increase of LFP theta frequency with running speed but for vertical movements only.

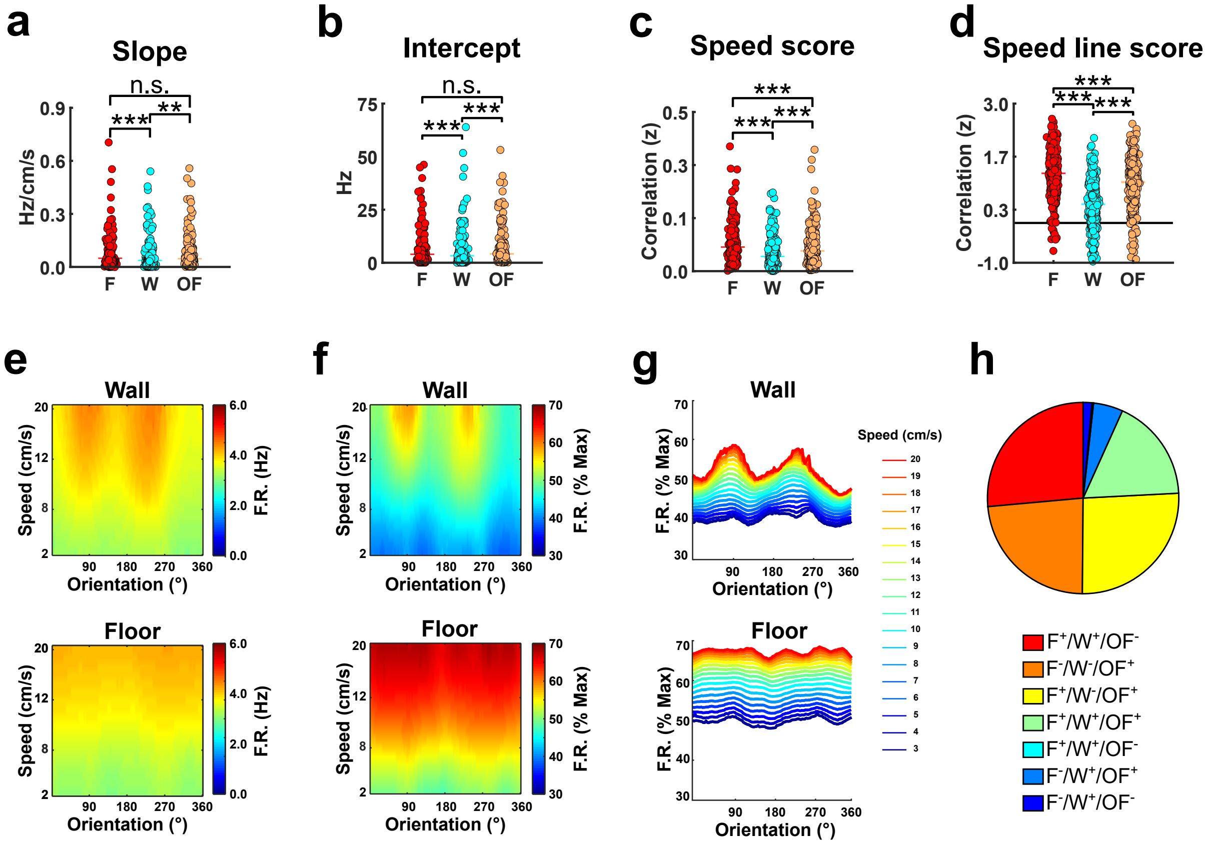

Fig. S8. Speed cell properties between surfaces.

401 of the total 461 speed cells were recorded in all three environments. Plots show individual cell values plus mean and s.e.m., * = $P < 0.05$, * * = $P < 0.01$, *** = $P < 0.001$.

The reduction in the firing rate of speed cells on the wall was due to both reduced intercept (**a**; floor = 4.0 ± 0.4 Hz, wall = 3.3 ± 0.3 Hz, open field = 4.2 ± 0.4 Hz, $F_{2,800}$ = 21.1, $P = 1.14e^{-09}$) and slope (**b**; floor = 0.049 ± 0.004 Hz/cm/s, wall = 0.037 ± 0.003 Hz/cm/s, open field = 0.046 ± 0.004 Hz/cm/s, $F_{2,800}$ = 10.7, $P = 2.56e^{-05}$) of the speed-firing rate relationship. On the wall speed cells displayed reduced speed score (**c**; floor = 0.068 ± 0.002, wall = 0.041 ± 0.002, open field = 0.057 ± 0.002; z-fisher transformation: $F_{2,800}$ = 102.7, $P = 1.31e^{-24}$) and speed line score (**d**; floor = 0.78 ± 0.01, wall = 0.37 ± 0.02, open field = 0.69 ± 0.02; z-fisher transformation*:* $F_{2,800}$ = 326.0, $P = 1.59e^{-22}$).

The observed reduction in speed cell firing rate on the wall prompted us to further explore potential effects of heading direction between surfaces. **e**; Mean firing rate of speed cells between surfaces (wall = top, floor = bottom) across heading direction (x-axis) and running speed (y-axis) represented as heat map (blue = 0.0 Hz, red = 8.0 Hz). Note the gradual increase in firing rate along running speed but not heading direction on the floor. In contrast, speed cell firing rate on the wall was generally reduced and showed increase only along upwards (90°) and downwards (270°) movements. **f**; speed cell firing rate between surfaces is shown by means of a 2D heat map, scaled as the percentage of

the peak firing rate on the floor for each individual session. On the floor there was a gradual increase along running speed only, whereas on the wall, the increase was only present along vertical movements. **g**; Speed cell firing rate shown by lines representing the percentage of the peak firing rate on the floor (y-axis) plotted along heading directions (x-axis) for each individual color-coded speed (low speed = dark blue, high speed = red). On the floor, the lines showed little directional modulation, in contrast to the wall where there was a clear increase in firing rate with running speed but for vertical movements only. **h,** Pie chart representing percentages of speed cells classed as significant on each surface: $F^+W^- O^-$ = 106 cells, $F^-W^-O^+$ = 94 cells, $F^+W^-O^+$ = 104 cells, $F^+W^+O^+$ = 70 cells, $F^+W^+O^-$ = 20 cells, $F^-W^+O^+$ = 1 cells, $F^-W^+O^-$ = 6 cells.

To account for possible bias in speed cell detection due to differences in directional sampling between surfaces, we also downsampled the data, as follows. For all the sessions where a speed cell was detected, the heading direction of the animal was first binned into 6° directional bins of both surfaces. For each bin, the dwell time on the floor was then undersampled to match the wall one by randomly removing the extra dwell time on the floor. Speed cell detection analyses were then conducted for each surface independently. This process was repeated 100 times for each cell, so that in each iteration a different set of data (but in equal number compared to the wall) could be analyzed. Once the random downsampling procedure was completed, the probability that a cell was previously selected as speed cell due to uneven sampling between floor and wall was calculated as follows:

$$P = \frac{1 - N\ significant\ iterations}{N\ iterations}$$

1For instance, for a cell passing criteria 99 times, the probability that it was classed as speed cell due to uneven heading bias on a given surface was 0.01. A cell was then scored as significant on each surface, if the resulting number was lower than 0.05, meaning that it had $P < 0.05$ to be classified as significant speed cell on that surface due to biases in heading directions on each surface. This analysis confirmed that 438/461 (> 95 %) cells passed speed cell acceptance criteria even after exhaustive downsampling - therefore validating our previous speed cell detection method. Moreover, the McNemar's test re-applied to this set of data confirmed a dramatic drop in the number of cells passing criteria on the wall compared to the floor (floor = 299, wall = 153, $\chi^2 = 106.3$, $P < 0.000001$).

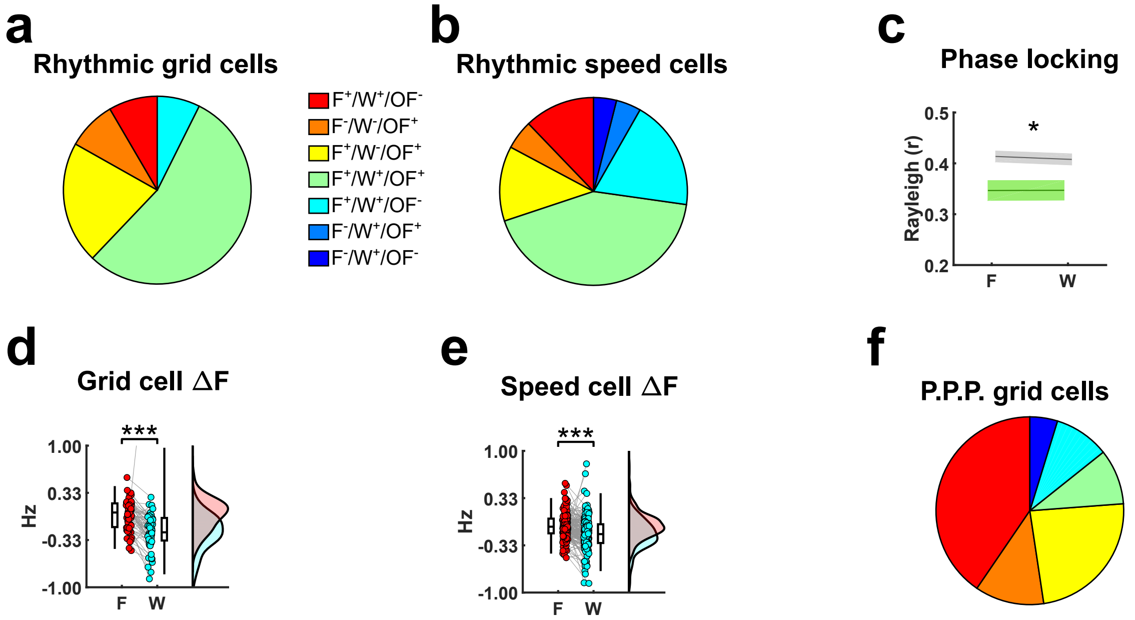

Fig. S9. Grid cell and speed cell spike train theta frequency across surfaces. **a**, Pie chart representing percentages of grid cells classed as significantly rhythmic on each surface: $F^+W^-O^-$ = 4 cells, $F^-W^-O^+$ = 8 cells, $F^+W^-O^+$ = 20 cells, $F^+W^+O^+$ = 52 cells, $F^+W^+O^-$ = 7 cells, $F^-W^+O^+$ = 0 cells, $F^-W^+O^-$ = 0 cells; floor = 87/95, wall = 59/95, McNemar's test; χ^2= 26.0, P = 3.35e^{-07}).

b, Pie chart representing percentages of speed cells classed as significantly rhythmic on each surface: $F^+W^-O^-$ = 119 cells, $F^-W^-O^+$ = 11 cells, $F^+W^-O^+$ = 36 cells, $F^+W^+O^+$ = 34 cells, $F^+W^+O^-$ = 14 cells, $F^-W^+O^+$ = 53 cells, $F^-W^+O^-$ = 12 cells; floor = 242/279, wall = 193/279, McNemar's test; χ^2= 25.3, P = 4.86e^{-07}).

c, Theta phase locking showed no changes between surfaces ($F_{1,229}$ = 0.373, P = 0.54), but was significantly decreased in grid cells compared to speed cells ($F_{1,229}$ = 8.64, P =0.0036) and there was no interaction ($F_{1,229}$ = 0.51, P = 0.48).

The difference between mean spike train theta frequency and LFP theta frequency (ΔF) was significantly reduced on the wall for both grid cells (**d,** floor = 0.023 ± 0.027 Hz, wall = -0.176 ± 0.054 Hz, t_{58} = 3.58, P = 0.00072) and speed cells (**e**; floor = -0.057 ± 0.014 Hz, wall = -0.168 ± 0.018 Hz, t_{171} = 6.01, P = 1.07e^{-08}).

f, Pie chart representing percentages of grid cells classed as PPP (putative phase precessing) on each surface: $F^+W^-O^-$ = 17 cells, $F^-W^-O^+$ = 5 cells, $F^+W^-O^+$ = 10 cells, $F^+W^+O^+$ = 4 cells, $F^+W^+O^-$ = 4 cells, $F^-W^+O^+$ = 0 cells, $F^-W^+O^-$ = 2 cells.

https://www.youtube.com/watch?v=JOqYTomi63w&feature=youtu.be
Movie S1. Video of rat behaviour in the enriched-housing environment.
